# Modelling-informed cell-seeded nerve repair construct designs for treating peripheral nerve injuries

**DOI:** 10.1371/journal.pcbi.1009142

**Published:** 2021-07-08

**Authors:** Rachel Coy, Maxime Berg, James B. Phillips, Rebecca J. Shipley

**Affiliations:** 1 Department of Mechanical Engineering, UCL, London, United Kingdom; 2 Center for Nerve Engineering, UCL, London, United Kingdom; 3 Department of Pharmacology, School of Pharmacy, UCL, London, United Kingdom; Northeastern University, UNITED STATES

## Abstract

Millions of people worldwide are affected by peripheral nerve injuries (PNI), involving billions of dollars in healthcare costs. Common outcomes for patients include paralysis and loss of sensation, often leading to lifelong pain and disability. Engineered Neural Tissue (EngNT) is being developed as an alternative to the current treatments for large-gap PNIs that show underwhelming functional recovery in many cases. EngNT repair constructs are composed of a stabilised hydrogel cylinder, surrounded by a sheath of material, to mimic the properties of nerve tissue. The technology also enables the spatial seeding of therapeutic cells in the hydrogel to promote nerve regeneration. The identification of mechanisms leading to maximal nerve regeneration and to functional recovery is a central challenge in the design of EngNT repair constructs. Using *in vivo* experiments in isolation is costly and time-consuming, offering a limited insight on the mechanisms underlying the performance of a given repair construct. To bridge this gap, we derive a cell-solute model and apply it to the case of EngNT repair constructs seeded with therapeutic cells which produce vascular endothelial growth factor (VEGF) under low oxygen conditions to promote vascularisation in the construct. The model comprises a set of coupled non-linear diffusion-reaction equations describing the evolving cell population along with its interactions with oxygen and VEGF fields during the first 24h after transplant into the nerve injury site. This model allows us to evaluate a wide range of repair construct designs (e.g. cell-seeding strategy, sheath material, culture conditions), the idea being that designs performing well over a short timescale could be shortlisted for *in vivo* trials. In particular, our results suggest that seeding cells beyond a certain density threshold is detrimental regardless of the situation considered, opening new avenues for future nerve tissue engineering.

## Author summary

Mathematical models are increasingly gaining traction in biomedical sciences in general and in peripheral nerve repair in particular. These models can both help to unveil mechanisms underlying nerve regeneration post-injury and inform the design of new repair strategies, while continuously being improved through experiments and clinical data. In particular, cell-solute models provide flexible, computationally-efficient frameworks that enable access to spatio-temporal information difficult to reach *in vivo* in nerve repair scenarios. In this work, we derive such a model and apply it to the case of Engineered Neural Tissue repair constructs after their transplant into the nerve injury site. One key feature of such repair constructs is the spatial seeding of therapeutic cells capable of producing signalling molecules that promote nerve regeneration. We simulate the activity of such cells and evaluate, after 24h, cell survival and signalling molecule output for a range of nerve repair construct designs. The underlying hypothesis is that designs performing well at such short timescales could be shortlisted for *in vivo* trials, the latter being costly and time-consuming. Our results suggest that seeding cells beyond a certain density threshold is detrimental regardless of the situation considered, opening new avenues for future nerve repair construct design.

## Introduction

More than one million people per year are affected by peripheral nerve injuries (PNI) in Europe and the USA [1] and healthcare costs are estimated to exceed £1Bn per year in the USA alone [2]. Paralysis and loss of sensation are hallmarks of severe PNIs, which can lead to lifelong pain and disability for patients. Neurons in the peripheral nervous system have some ability to regenerate following damage, via a process involving both neuronal and non-neuronal cells and signalling molecules.

The extent of nerve regeneration is largely determined by the severity of the injury, which often requires a surgical intervention. Gold-standard treatments for large-gap PNIs involve an autograft, *i*.*e*. the transplantation of healthy nerve sections from elsewhere in a patient’s body into the injury-induced nerve gap. However, studies have reported that only around 50% of autograft patients regain a good level of function [3–6]. In addition, nerve and tissue damage at the donor nerve site results in scarring and loss of function and can lead to more pain and increased risk of infection. The need to harvest a donor nerve also lengthens the time spent in surgery and the number of eligible nerve donor sites is limited.

Bioengineered peripheral Nerve Repair Constructs (NRCs) are being developed as alternatives to address these issues. A large range of synthetic and natural biomaterial bases for these constructs have been explored [7,8]; here we focus on one example with high translational potential, Engineered Neural Tissue (EngNT) [9]. EngNT constructs are composed of stabilised hydrogel, typically collagen, containing aligned therapeutic cells. Many cell types have been explored dependent on the therapeutic scenario [10] including differentiated adipose-derived stem cells (dADSC) that are able to produce Vascular Endothelial Growth factor (VEGF) under low oxygen conditions [11,12] to promote revascularisation of the injury site. EngNT constructs mimic the geometry of the host nerve, so are typically cylindrical in shape, and surrounded by a protective sheath of material that can be made permeable to exchange of molecules. These NRCs provide a supportive microenvironment to promote neurite and blood vessel regrowth between nerve stumps, and the technology potentially enables careful spatial seeding of therapeutic cells to facilitate this [13].

In this context, the goal is then to determine the design of NRCs that would yield maximal nerve regeneration and lead to functional recovery. So far, most of the investigations on this question have generally focused on experimentally measuring the benefit of a given NRC design after several days to several weeks after transplant (*e*.*g*. therapeutic cell type, sheath material, see references in [10,14]), which is costly and time-consuming. On the other hand, short-term impacts of cell-seeding strategies, culture conditions (*e*.*g*. initial concentration of oxygen) or sheath material (*e*.*g*. sheath porosity) on cell survival and signalling factor production, which are likely to affect long-term regeneration, are challenging to assess experimentally *in vivo* because there are so many possible options to test.

Mathematical models have been widely used in the field of regenerative medicine [15], including peripheral nerve repair [16]. They generally provide a description of the interactions between cells and their environment at different scales, including the surrounding substrate, nutrients and signalling factors. For problems that require a description of subcellular mechanisms, discrete models such as Agent-Based Model [17] have been developed to retrieve collective cell behaviours, including tumour [18] and soft tissue growth [19].

For problems requiring a description at the scale of the whole tissue, models based on multiphase theory in porous media [20,21] have been developed. These models consider the cells and their surrounding microenvironment to be a continuum with effective properties. In particular, these models have been used to describe situations analogous to EngNT constructs in the context of tissue culture bioreactors [22–27].

Broadly speaking, these models comprise a set of effective, non-linear coupled partial differential equations describing both the spatial and the temporal behaviours of cell populations, solute transport and the porous media formed by the substrate (e.g., structured scaffold, hydrogel). For instance, Lemon et al.[28] developed a 3-phase model (i.e., cell, culture media, porous scaffold) with a focus on mechanical interactions, allowing them to describe cell migration in an engineered tissue. This model was later refined to include cell proliferation and nutrient transport [29,30] making it a general framework to study engineered tissue. Similarly, Ochoa et al.[31] used formal Volume Averaging [21], to derive a model of solute transport in an idealized cellular media and were able to obtain representative transport equations at the scale of the whole media. Such a model was then extended to biofilms [32] and to culture bioreactors [33,34], taking into account cell metabolism and cell proliferation, making it a strong candidate for cell-solute descriptions for engineered tissue.

Continuous models can therefore be seen as valuable tools to study engineered tissue at a limited cost. That is why in the present work, given the typical size of NRC constructs (>1cm), the number of cells usually seeded (between 3×10^4^ and 1.2×10^6^ [35,36]) and the time scale we are interested in (24h), we derive a continuous model focusing on cell-solute interactions in an EngNT seeded with dADSCs, since the latter are relevant for NRCs currently under development and have been characterised *in vitro* [12]. We point out that, even though we focus on specific types of constructs and therapeutic cells, we derive the cell-solute model in a relatively general manner that can easily be extended to other repair scenarios. The objective of such a model is then to assess the performance of different NRC designs within the first 24h following transplant.

Briefly, during this period of time the therapeutic cells proliferate in the collagen, consuming the oxygen present in their environment. Then, when the oxygen concentration reaches low values, the cells become stressed and produce VEGF that will later promote the migration of endothelial cells and the creation of a vascular network that will perfuse the construct with oxygen and nutrients, supporting both the therapeutic cell population and the subsequent neurite regrowth. During the first 24h, we therefore focus on simulating the evolving cell population, oxygen, and VEGF concentrations, along with their interactions, before endothelial cell migration occurs. We then determine which design yields the maximal mean cell density, as therapeutic cells, besides VEGF secretion, can also provide mechanical support via the production of extra cellular matrix proteins [37,38] and directional cues [9] for vessel sprouts and neurites. We also determine which design yields the maximal mean VEGF concentration and maximal VEGF gradients after 24h, as VEGF plays a central role in regenerative angiogenesis, which is involved in subsequent stages of nerve repair [39].

In doing so, the underlying ideas are that 1) designs that perform poorly on this short-time scale could be discarded, hence saving experimental time and 2) modelling could help us understand the underlying mechanisms influencing performance and could indicate new avenues for design improvement.

The model itself is parametrised using *in vitro* data when possible and left uncertain otherwise. Doing so allows us to evaluate in a systematic manner a wide range of NRC designs. In particular, we investigate the impact of different cell-seeding strategies (initial cell density and initial spatial distribution of cells), as therapeutic cell distribution is likely to control VEGF secretion and cell survival. We also investigate the influence of sheath material (*e*.*g*., porosity, thickness), culture conditions pre-implantation (*e*.*g*. initial oxygen concentration) and surrounding tissue conditions, on cell survival and vascular growth factor production.

## Methods

### Nerve repair construct geometry

**Fig 1:**
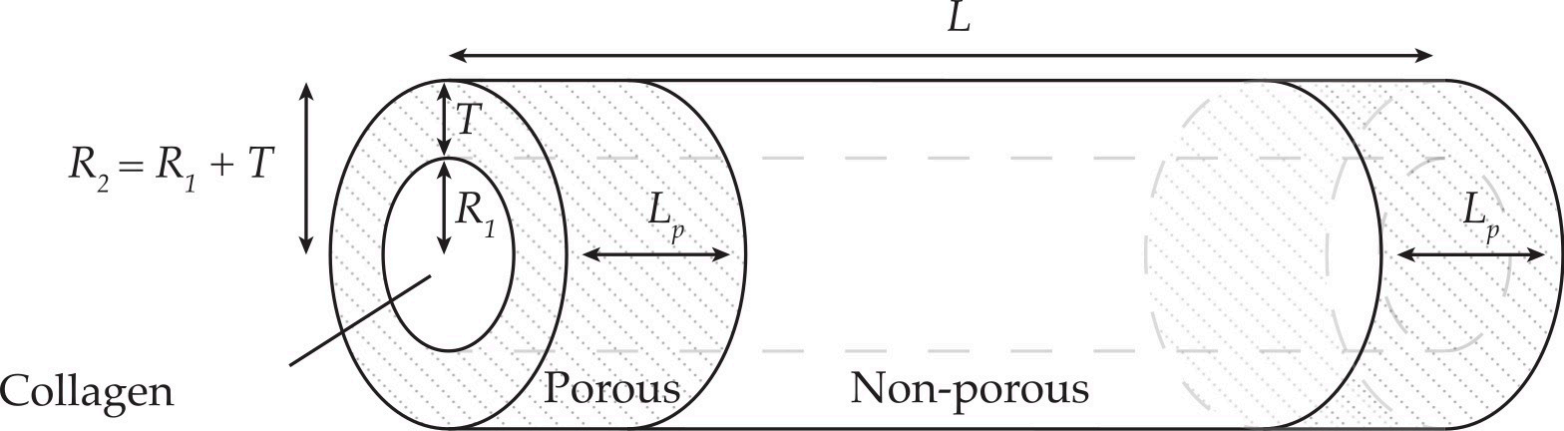
Nerve repair construct geometry. Sheath partially made of porous material permeable to molecular exchanges at the construct extremities. The parameter *R*_1_ denotes the radius of the cell-seeded collagen cylindrical core, *T* the thickness of the sheath, *R*_2_ = *R*_1_+*T* the radius of the nerve repair construct, *L* the length of the construct and *L*_*p*_ the length of the porous section of the sheath (2*L*_*p*_ in total).

We consider the NRC to be composed of a cell-seeded collagen cylindrical core of length *L* (m) and radius *R*_1_ (m) surrounded by an annular, acellular sheath of materials of thickness *T* (m), as illustrated in Fig 1. This geometry mimics a typical repair scenario in an *in vivo* test model, such as the rat sciatic nerve. The sheath itself can be impermeable (Fig 1 when *L*_*p*_ = 0), partially or entirely (Fig 1 when 2*L*_*p*_ = *L*) permeable to molecular exchanges with the surrounding tissue. We further assume that the core and the sheath form two distinct continuous media with properties that can be described using an effective description.

### Cell density dynamics

Different therapeutic cell types have been used in EngNT designs [13,40]. As mentioned in the Introduction, we focus on the case of dADSCs because they are relevant for EngNT NRCs currently under development and have been characterised *in vitro*. However, the cell-solute model is derived in a relatively generic manner and could be readily adapted for other cell types.

In general, the local cell density is determined by the growth and decay rates of the cell population, as well as cell migration. In stabilised collagen gels, however, cell migration can take weeks, and *in vitro* experiments using different cells in similar materials have shown no significant cell displacements over the first five days following the seeding [41]. Given that we focus on the first 24 hours post implantation, in this work we neglect cell migration mechanisms and write the following balance for the local cell density

$$\partial_{t}n=G_{n}-Q_{n},$$ (1)

where *n* represents the local cell density (cell∙m^-3^), *G*_*n*_ the cell density growth (cell∙m^-3^∙s^-1^), and *Q*_*n*_ the cell density decay (cell∙m^-3^∙s^-1^). Following the work of Coy et al. [12], we assume that cell density growth has the following form

$$G_{n}=G_{n}(n,c_{g})=\beta c_{g}n\left(1-\frac{n}{n_{\max}}\right),$$ (2)

where *c*_g_ denotes the local oxygen concentration in the gel (kg∙m^-3^), *β* the cell growth rate (m^3^∙kg^-1^∙s^-1^) and *n*_max_ the maximum cell density (cell∙m^-3^). We note that the cell growth term depends only on local cell density and oxygen concentration since we have assumed all other substrates, such as glucose, to be in excess. We also note that the local cell density follows a logistic growth form to account for nutrient and space competition, hence the inclusion of *n*_max_. With regards to cell decay we also follow the work of Coy et al. [12] who assumed a linear decay term, yielding

$$Q_{n}=Q_{n}(n)=\delta n,$$ (3)

where *δ* denotes the cell death rate (s^-1^). We note that *δ* is in fact an increasing linear function of the initial cell density to represent the effects of initial crowding on later time points and provide its expression in the parameter value section below.

### Molecular transport in the cell-seeded collagen gel

PNI regeneration involves a large range of molecules that interact with each other and the embedded cells. In this work, however, we focus on oxygen and vascular growth factors, as these molecules along with their interactions are central in cell survival and angiogenesis. Other nutrients such as glucose are assumed to be in excess for the first 24h, and the impact of other growth factors such as brain derived neurotrophic factor, glial cell line-derived neurotrophic factor or nerve growth factor are neglected. However, the framework we establish is sufficiently general that these molecules could be included in future studies.

In this context, we consider the transport of oxygen to be driven by molecular diffusion and assume that cells metabolize oxygen following Michaelis-Menten kinetics, as is commonly used in the literature for oxygen-limited conditions [42–45], so that

$$\partial_{t}c_{g}=D_{c,g}\nabla^{2}c_{g}-nM\frac{c_{g}}{c_{1/2}+c_{g}},$$ (4)

where *c*_g_ corresponds to the local oxygen concentration (kg∙m^-3^), *D*_*c*,g_ to the diffusion coefficient of oxygen within the collagen gel (m^2^∙s^-1^), *M* to the maximal metabolic rate (kg∙cell^-1^∙s^-1^) and *c*_1/2_ to the oxygen concentration (kg∙m^-3^) for which the metabolic reaction equals half its maximal value.

Vascular growth factors form a family of molecules. In this work, however, we focus on modelling vascular endothelial growth factor-A (VEGF-A), which is regarded as the most influential molecule of the VEGF family upon angiogenesis [46,47]. In the following, VEGF-A is simply referred to as VEGF for simplicity.

In this context we assume the transport of VEGF in the cell-seeded collagen gel to be driven by molecular diffusion. Contrarily to oxygen, which is a stable molecule, VEGF, which is secreted by the cells, is an unstable protein and its degradation is usually modelled using a linear decay term [48–52] so that

$$\partial_{t}v_{g}=D_{v,g}\nabla^{2}v_{g}+G_{v}-Kv_{g},$$ (5)

where *v*_g_ corresponds to the local VEGF concentration (kg∙m^-3^), *D*_*v*,g_ to the diffusion coefficient of VEGF within the collagen gel (m^2^∙s^-1^), *K* to the VEGF degradation rate (s^−1^) and *G*_*v*_(*n*,*c*) to the production rate (kg∙m^-3^∙s^-1^) of VEGF. With regards to VEGF production, we use the expression derived by Coy et al. [12], which takes into account upregulation of VEGF in low-oxygen transport regimes so that

$${G_{v}=G}_{v}(n,c_{g})=\alpha n\left(\frac{V_{m}+1}{2}-\frac{V_{m}-1}{2}\tanh(B(c_{g}-c_{h}))\right),$$ (6)

where *α* denotes the baseline VEGF production rate (kg∙cell^-1^∙s^-1^), *c*_*h*_ the oxygen threshold under which VEGF production is upregulated by oxygen (kg∙m^-3^), *V*_*m*_ the factor controlling the magnitude of such a regulation and *B* (m^3^∙kg^-1^) the factor controlling the transition between baseline and regulated states. We point out that both *α* and *V*_*m*_ are in fact functions of the initial cell density, and that their expressions are given in the parameter value section below.

Finally, we note that, in general, the diffusion coefficients in Eqs 4 and 5 should depend on the cell density, as an increase in cell density implies a higher tortuosity in the gel and anomalous diffusion due to interfacial effects. Practically however, this yields a significant impact only for high cell densities [44,53], which are not the focus of this work. Analogous to cell migration mechanisms, we also neglect effects of collagen matrix remodelling on molecular transport, assuming that it takes place on a longer timescale. Hence, we assume the diffusion coefficients to be independent of the cell density in the gel and the evolving collagen structure.

### Molecular transport in the annular sheath

We consider the sheath surrounding the gel to be a porous media devoid of any cells and its porosity to be a free parameter ranging from 0 (completely impermeable to molecular exchanges) to 1 (completely permeable). We further consider the pores to be larger than the characteristic size of the molecules transported. In this situation we can no longer neglect *a priori* the effects of porosity and tortuosity on molecular transport [54]. Therefore, the transport equations for oxygen and VEGF in the sheath are given by

$$\epsilon \partial_{t}c_{s}=D_{c,s}\frac{\epsilon }{\tau (\epsilon )}\nabla^{2}c_{s},$$ (7)

for oxygen and

$$\epsilon \partial_{t}v_{s}=D_{v,s}\frac{\epsilon }{\tau (\epsilon )}\nabla^{2}v_{s}-K\epsilon v_{s},$$ (8)

for VEGF, where *D*_*c*,s_ and *D*_*v*,s_ denote the molecular diffusion coefficient of oxygen and VEGF respectively (m^2^∙s^-1^) in the sheath, *ϵ* denotes the sheath porosity and *τ* its tortuosity. We recall that the tortuosity is a function of the underlying porous structure, which varies widely according to the materials and methods used to create the NRC sheath [55–57]. In this work we propose to take a step back and narrow our focus to archetypal structures, commonly encountered in porous media, for which it is possible to derive theoretically an expression for the tortuosity. Precisely, we will consider the cases of overlapping spheres [58], cylinders [59] and disordered tubes [60]. Table 1 summarizes the different expressions for the tortuosity depending on the aforementioned porous structures.

**Table 1:** Tortuosity expressions as a function of porous structures.

Porous Structure	Tortuosity Description (*τ*(*ϵ*))
Overlapping Spheres [58]	$1-\frac{1}{2}ln(\epsilon )$
Cylinders [59]	$\frac{3\epsilon }{4\epsilon -1}$
Disordered Tubes [60]	$\frac{\epsilon }{1-{\left(1-\epsilon \right)}^{1/3}}$

### Initial and boundary conditions

Starting with the envelope of the NRC, we expect the tissue surrounding it to maintain, at least during the first days after the graft, a form of homeostasis and therefore assume that both oxygen and VEGF fields are constant outside of the construct. We note that, practically, concentration values at the severed nerve ends and in the surrounding tissue are likely to be different. Due to the lack of measures however, we set them at a value, representative of nerve tissue in general. This translates into the following Dirichlet conditions

$$c_{g}(r,\theta ,Z,t)=c_{\mathrm{tissue}};Z\in \{0,L\},$$ (9)

$$v_{g}(r,\theta ,Z,t)=v_{\mathrm{tissue}};\;Z\in \{0,L\},$$ (10)

for the oxygen and VEGF concentration fields associated to the gel and

$$c_{s}(r,\theta ,Z,t)=c_{\mathrm{tissue}};Z\in \{0,L\},$$ (11)

$$v_{s}(r,\theta ,Z,t)=v_{\mathrm{tissue}};\;Z\in \{0,L\},$$ (12)

$$c_{s}(R_{2},\theta ,z,t)=c_{\mathrm{tissue}},$$ (13)

$$v_{s}(R_{2},\theta ,z,t)=v_{\mathrm{tissue}},$$ (14)

for the oxygen and VEGF concentration fields associated to the annular sheath.

Next, with regards to the internal boundaries, we impose conservation of the molecular flux at the interface between the cylinder core and the sheath (*r* = *R*_1_) for both the oxygen and VEGF fields

$$D_{c,g}\partial_{r}c_{g}(R_{1},\theta ,z,t)=D_{c,s}\frac{\epsilon }{\tau (\epsilon )}\partial_{r}c_{s}(R_{1},\theta ,z,t),$$ (15)

$$D_{v,g}\partial_{r}v_{g}(R_{1},\theta ,z,t)=D_{v,s}\frac{\epsilon }{\tau (\epsilon )}\partial_{r}v_{s}(R_{1},\theta ,z,t).$$ (16)

In addition, we assume that the diffusion process is slow enough so that the local thermodynamic equilibrium is reached at the interface between gel and sheath material yielding

$$\underset{t\to \infty }{\lim}c_{g}(R_{1},\theta ,z,t)-\lambda_{c}c_{s}(R_{1},\theta ,z,t)=0,$$ (17)

$$\underset{t\to \infty }{\lim}v_{g}(R_{1},\theta ,z,t)-\lambda_{v}c_{s}(R_{1},\theta ,z,t)=0,$$ (18)

where *λ*_*c*_ and *λ*_*v*_ denote the partition coefficients between the gel and the sheath for the oxygen and VEGF respectively. We note that there is no need for boundary conditions for the cell density since we assumed no migration mechanisms.

With regards to initial conditions, we prescribe a uniform concentration throughout the construct for both oxygen and VEGF to represent storage conditions before implantation into the injury site, so that

$$c_{i}(r,\theta ,z,0)=c_{0};i\in \{g,s\},$$ (19)

$$v_{i}(r,\theta ,z,0)=v_{0};i\in \{g,s\}.$$ (20)

Finally, we impose the initial cell density to be uniform across the cylinder core cross-section but still able to depend upon axial position, yielding

$$n(r,\theta ,z,0)=n_{0}(z),$$ (21)

leaving the opportunity to explore the consequences of non-uniform initial cell-seeding on oxygen and VEGF distributions.

### Cell seeding strategy evaluation

One of the main aims of this work is to identify cell seeding strategies that could increase cell density, along with VEGF concentrations and gradients within NRCs that may favour vascular regrowth. To that end, we first define the following quantities in the cylindrical core

$$\bar{n}(t)=\frac{1}{\pi R_{1}^{2}L}{∭}_{V}n(r,\theta ,z,t)dV,$$ (22)

$$\bar{c}(t)=\frac{1}{\pi R_{1}^{2}L}{∭}_{V}c(r,\theta ,z,t)dV,$$ (23)

$$\bar{v}(t)=\frac{1}{\pi R_{1}^{2}L}{∭}_{V}v(r,\theta ,z,t)dV,$$ (24)

$$v_{SD}^{2}(t)=\frac{1}{\pi R_{1}^{2}L}{∭}_{V}{\left(v(r,\theta ,z,t)-\bar{v}\right)}^{2}dV,$$ (25)

where $\bar{n}$ denotes the average cell density, $\bar{c}$ the average oxygen concentration, $\bar{v}$ the average VEGF concentration over *V*, the volume of the collagen core. *v*_SD_ represents the standard deviation associated to the VEGF concentration through the collagen core. *v*_SD_ is used here an indirect measure of VEGF gradients. We then define initial cell densities that will maximise $\bar{n},\;\bar{v}$ and *v*_SD_ the following way

$$\mathrm{argma}x_{n_{0}}\bar{n}(t)=\{n_{0}|\bar{n}=\underset{n_{0}}{\max}\bar{n}\prime \},$$ (26)

$$\mathrm{argma}x_{n_{0}}\bar{v}(t)=\{n_{0}|\bar{v}=\underset{n_{0}}{\max}\bar{v}\prime \},$$ (27)

$$\mathrm{argma}x_{n_{0}}v_{SD}^{}(t)=\{n_{0}|v_{SD}^{}=\underset{n_{0}}{\max}v_{SD}^{\prime }\},$$ (28)

where $\mathrm{argma}x_{n_{0}}\bar{n},\;\mathrm{argma}x_{n_{0}}\bar{v}$ and $\mathrm{argma}x_{n_{0}}v_{SD}^{}$ represent the initial cell densities leading to the maximal average cell density, maximal average VEGF concentration and maximal VEGF spreading at time *t* respectively.

### Simulations

Simulations of different cell densities and distributions over a NRC geometry along with prediction of oxygen and VEGF concentrations are carried out using finite element methods using COMSOL Multiphysics.

### Parameter values

The parameters associated with this problem can be divided into two classes. First there are the parameters that have been previously determined using experiments, such as those associated with the transport of oxygen and VEGF in the collagen gel [12]. Then, there are parameters for which values are rather taken from a range, whether due to lack of contextual information (*e*.*g*. VEGF and oxygen concentrations in the tissue surrounding the NRC) or due to deliberate design choices (*e*.*g*. initial cell densities, sheath thickness). In the following tables, parameters are given defined values when possible and ranges of values otherwise. We also point out that Tables 2–6 present parameter values in two different systems of units; practical units, *i*.*e*. units useful for discussing and disseminating results (*e*.*g*. concentrations in %O_2_, ng∙ml^-1^), and modelling units, *i*.*e*. SI units useful for solving mass balance equations (*e*.*g*. concentrations in kg∙m^-3^).

**Table 2:** NRC geometry parameters.

	Practical units	Modelling units
NRC length (*L*)	15 mm	1.50×10^−2^ m
Cylinder core radius (*R*_1_)	0.25 mm	2.50×10^−4^ m
Sheath thickness (*T*)	[0.25, 1.50] mm	[2.50×10^-4^, 1.50×10^-3^] m
NRC radius (*R*_2_ = *R*_1_+*T*)	[0.50, 1.75] mm	[5.00×10^-4^, 1.75×10^-3^] m
Porosity (*ϵ*)	[0,1]	[0, 1]

**Table 3:** Cell density dynamics parameters.

	Practical units	Modelling units	Ref
Cell growth rate (*β*)	220 ml∙mol^-1^∙s^-1^	7.10×10^-3^m^3^∙kg^-1^∙s^-1^	[12]
Max. cell density (*n*_max_)	4.00×10^8^cell∙ml^-1^	4.00×10^14^cell∙m^-3^	[12]
Cell decay rate (*δ*_0_)	1.13×10^-5^s^-1^	1.13×10^-5^s^-1^	[12]
Cell decay rate (*δ*_1_)	9.13×10^-14^ml∙cell^−1^∙s^-1^	9.13×10^-23^m^3^∙cell^−1^∙s^-1^	[12]

**Table 4:** Oxygen transport parameters.

	Practical units	Modelling units	Ref.
Diffusion coef. in gel (*D*_*c*,g_)	4.50×10^-6^cm^2^∙s^-1^	4.50×10^-10^m^2^∙s^-1^	[67]
Max. metabolic rate (*M*)	2.00×10^-19^mol∙cell^-1^∙s^-1^	6.20×10^-21^kg∙cell^-1^∙s^-1^	[12]
½ max. con. (*c*_1/2_)	6.67×10^-9^mol∙ml^-1^ 0.51% O_2_	2.07×10^-4^kg∙m^-3^	[12]
Diffusion coef. in sheath (*D*_*c*,s_)	2.63×10^-5^cm^2^∙s^-1^	2.63×10^-9^m^2^∙s^-1^	[68]

**Table 5:** VEGF transport parameters.

	Practical units	Modelling units	Ref.
Diffusion coef. gel (*D*_*v*,g_)	1.13×10^-6^cm^2^∙s^-1^	1.13×10^-10^m^2^∙s^-1^	[12]
VEGF prod. rate (*α*_0_)	4.60×10^-25^mol∙cell^-1∙^s^-1^ 2.17×10^-11^ng∙cell^-1^∙s^-1^	2.17×10^-23^kg∙cell^-1^∙s^-1^	[12]
VEGF prod. rate (*α*_1_)	6.73×10^-34^mol∙ml∙cell^-2^∙s^-1^ 3.10×10^-20^ng∙ml∙cell^-2^∙s^-1^	3.10×10^-38^kg∙m^3^∙cell^-2^∙s^-1^	[12]
VEGF prod. rate (*α*_2_)	5.43×10^-42^mol∙ml^2^∙cell^-3^∙s^-1^ 2.60×10^-28^ng∙ml^2^∙cell^-3^∙s^-1^	2.60×10^-52^kg∙m^6^∙cell^-3^∙s^-1^	[12]
Regulation factor (*V*_*m*,0_)	5.22	5.22	[12]
Regulation factor (*V*_*m*,1_)	-9.04×10^-9^ml∙cell^-1^	-9.04×10^-15^m^3^∙cell^-1^	[12]
Transition factor (*B*)	6.77×10^9^ml^−1^∙mol $90{\%O}_{2}^{-1}$	4.35×10^5^m^3^∙kg^-1^	[12]
Hypoxia threshold (*c*_*h*_)	6.30×10^-8^mol∙ml^-1^ 4.77% O_2_	1.95×10^-3^kg∙m^-3^	[12]
Degradation rate (*K*)	2.99×10^-5^s^-1^	2.99×10^-5^s^-1^	[12]
Diffusion coef. sheath (*D*_*v*,s_)	1.37×10^-6^cm^2^∙s^-1^	1.37×10^-10^m^2^∙s^-1^	[69]

**Table 6:** Initial and boundary conditions parameters.

	Practical units	Modelling units
Tissue O2 con. (*c*_tissue_)	[1.32×10^-8^,1.32×10^-7^] mol∙ml^-1^ [1,10] %O_2_	[4.10×10^-4^,4.10×10^-3^] kg∙m^-3^
Tissue VEGF con. (*v*_tissue_)	[0,2.17×10^-14^] mol∙ml^-1^ [0,1] ng∙ml^-1^	[0,1.00×10^-6^] kg∙m^-3^
O2 partition coef. (*λ*_*c*_)	1	1
VEGF partition coef. (*λ*_*v*_)	1	1
Initial O2 con. (c_0_)	[1.32×10^-8^,2.78×10^-7^] mol∙ml^-1^ [1,21] %O2	[4.10×10^-4^,8.62×10^-3^] kg∙m^-3^
Initial VEGF con. (*v*_0_)	[0,2.17×10^-14^] mol∙ml^-1^ [0,1] ng∙ml^-1^	[0,1.00×10^-6^] kg∙m^-3^
Initial cell density (*n*_0_)	[1.00×10^7^,4.00×10^8^] cell∙ml^-1^	[1.00×10^13^,4.00×10^14^] cell∙m^-3^

Starting with the NRC itself, Table 2 summarizes the characteristics of the cylinder core and annular sheath geometry. We note that the choice for the NRC length *L* and cylinder radius *R*_1_ was made according to NRC designs currently under development. The range of values for the sheath thickness *T* was made to reflect existing designs [55,61–66].

Table 3 presents parameters associated with the cell population dynamics (Eqs 1–3). We recall that the cell death rate *δ* is a function of the initial cell density and has the following expression [12]

$$\delta =\delta_{0}+\delta_{1}n_{0},$$ (29)

where *δ*_0_ and *δ*_1_ are fitting parameters which values are reported in Table 3.

Tables 4 and 5 summarize the parameter values associated with the transport of oxygen and VEGF in the gel and the sheath (Eqs 4–8). We note that we consider the phase filling the pores of the sheath to be similar to water, and hence choose the value for the diffusion coefficient accordingly. We also recall that the baseline VEGF production rate *α* and the regulation magnitude factor *V*_*m*_ are functions of the initial cell density *n*_0_ and have the following expressions [12]

$$\alpha (n_{0})=\alpha_{0}+\alpha_{1}n_{0}+\alpha_{2}n_{0}^{2},$$ (30)

$$V_{m}(n_{0})=V_{m,0}+V_{m,1}n_{0},$$ (31)

where *α*_0_, *α*_1_, *α*_2_, *V*_*m*,0_ and *V*_*m*,1_ are fitting parameters with values are reported in Table 5.

Finally, Table 6 summarizes the initial and boundary condition values. We point out that due to the lack of oxygen concentration data available for the rat sciatic nerve model, the choice for the range of tissue oxygen concentration *c*_tissue_ was made to encompass the physiological oxygen range of 4–7% O_2_ measured *in vivo* in humans [70]. Similarly, the range of values for tissue VEGF concentration is based on measurements in serum (1.70×10^-2^ to 3.00×10^-1^ ng∙ml^-1^) and plasma (1.70×10^-2^ to 3.00*10^-1^ ng∙ml^-1^) of healthy controls [71]. We also note that the initial oxygen concentration ranges from 1% to 21%O_2_ to reflect possible storage conditions pre-implantation into the injury site, and that initial VEGF concentration ranges similarly to the tissue VEGF concentration for consistency. As for initial cell density, the range of values used encompasses the average *in vivo* Schwan cell density in normal nerve (2.00×10^7^ cell∙ml^-1^)[35,72], as well as typical cell densities used in NRCs (2.00×10^7^ to 1.60×10^8^ cell∙ml^-1^) [35,36]. Finally, following the work of Coy et al. [12] we assume that the partition coefficients *λ*_*c*_ and *λ*_*v*_ are close to one so that Eqs 17 and 18 simplify to continuity of the concentrations between gel and sheath.

## Results

We simulate cell-solute interactions in an array of nerve repair scenarios using the model derived in the method section. Each scenario has a different setting but the same structure; we seed an increasing number of cells in the collagen gel and determine which initial cell density yields the highest mean cell density ($\mathrm{argma}x_{n_{0}}\bar{n}$), highest mean VEGF concentration ($\mathrm{argma}x_{n_{0}}\bar{v}$) and largest standard deviation in VEGF concentration ($\mathrm{argma}x_{n_{0}}v_{SD}^{}$) 24h after transplant. We recall that $\mathrm{argma}x_{n_{0}}v_{SD}^{}$ is used as an indirect measure of VEGF gradient at the scale of the NRC. As mentioned in the Introduction, mean cell density, VEGF concentration and VEGF gradients are involved in regenerative angiogenesis which is one of the subsequent steps in nerve repair.

The scenarios are organised as follows. We start by assuming the sheath surrounding the collagen core to be impermeable and explore a range of storage conditions (*i*.*e*. initial oxygen and VEGF concentrations) and tissue conditions (i.e. oxygen and VEGF concentrations at the nerve stumps). We then estimate the effects of sheath porosity, sheath material (*i*.*e*. the underlying porous structure) and sheath thickness. Finally, we investigate the cases of partially porous sheaths (*i*.*e*. sheath with different porous length) and non-uniform seeding strategies (*e*.*g*. seeding cell preferentially in the centre of the construct or close to the nerve stumps).

In each scenario, unless specified, the initial concentration of oxygen in the collagen gel and sheath is equal to 21%O_2_, the surrounding tissue concentration of oxygen is equal to 5%O_2_ and both the initial and tissue VEGF concentrations are equal to zero. Further, when applicable, the sheath is assumed to have a globular structure that could be approximated by a body of overlapping spheres with an associated porosity *ϵ* = 0.8 and a thickness *T* = 0.25mm.

### Impermeable sheath

In this section we assume the sheath surrounding the gel to be impermeable so that molecular exchanges between the NRC and surrounding tissue only happen at the proximal and distal stumps. The underlying idea here is that an impermeable sheath could prevent the loss of VEGF to the tissue, hence increasing its concentration in the construct, with potential benefits in stimulating blood vessel growth.

**Fig 2:**
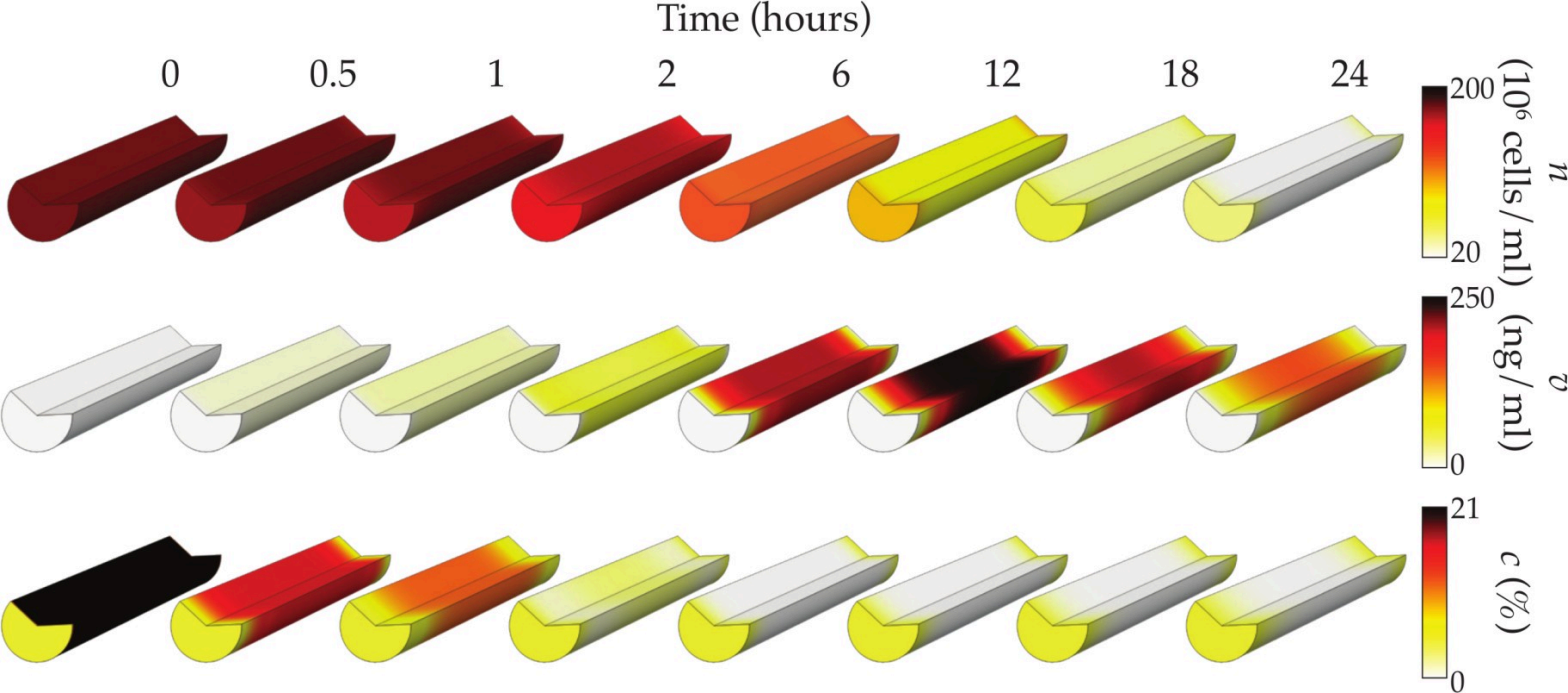
Cell-solute interactions in an impermeable nerve repair construct result in non-uniform distributions of cells, oxygen and VEGF. Heatmaps of cell density, oxygen and VEGF concentration over 24 hours for an initial cell density *n*_0_ = 1.78×10^8^ cells/ml. Only the collagen gel cylindrical core is displayed as the surrounding sheath is impermeable.


Fig 2 shows heatmaps of cell density, VEGF concentration and oxygen concentration over 24h for an initial uniform cell density *n*_0_ = 1.78×10^8^ cells/ml. The cell density globally decreases non-uniformly over 24 hours, generally with regions of higher cell density close to proximal and distal stumps. This global decrease is mainly due to the longitudinal diffusion time of oxygen

$$t_{c,L}=\frac{L^{2}}{{4D}_{c,g}},$$ (32)

being much longer (*t*_*c*,L_≈34.5h≈1.44 days) than the consumption time of oxygen (*t*_*c*,M_≈1.95h)

$$t_{c,M}=\frac{c_{0}}{Mn_{0}}.$$ (33)

This means that oxygen is consumed faster than it is delivered from the surrounding tissue. We note that at $t=0,\frac{dn}{dt}\geq 0$ (Eq 1), so that the cells are actually proliferating. This is due to the high initial concentration of oxygen, which can sustain cell proliferation on a short timescale. After a few hours, however, most of the initial oxygen has been consumed and new oxygen molecules have yet to diffuse from the tissue. This causes a significant decrease in the cell proliferation rate *G*_*n*_ (Eq 2), resulting in the global decrease observed in Fig 2.

With regards to the spatial non-uniformities, for the first 6 hours the cell density is lowest close to proximal and distal stumps. The oxygen concentration in these regions is lower than in the middle of the construct at early times due to the loss of oxygen to the tissue by diffusion (*c*_0_>*c*_tissue_). This lower concentration comparatively hinders cell proliferation, resulting in an earlier decrease in cell density. After 6h, however, cell densities are the lowest close to the centre of the construct. At this time, cells have consumed most of the oxygen initially present in the construct. Cells close to the proximal and distal stumps, however, have still access to oxygen from the tissue. In addition, cells close to the centre receive only a fraction of the oxygen coming from the tissue as it is first consumed by the cells close to the stumps. These inequalities in oxygen access result in the observed larger cell density close to the extremities and lower cell density in the centre.

Fig 2 also shows that the VEGF concentration increases for the first 12 hours and then decreases in a non-uniform manner. The initial increase is due to the longitudinal diffusion time of VEGF

$$t_{v,L}=\frac{L^{2}}{{4D}_{v,g}},$$ (34)

being much longer (*t*_*v*,L_≈139h≈5.8 days) than the secretion time of VEGF (*t*_*v*,P_≈3.63h) defined as

$$t_{v,P}=\frac{v_{p}}{\alpha (n_{0})n_{0}},$$ (35)

where *v*_*p*_ is a VEGF concentration (here *v*_*p*_ = 100 ng/ml, which corresponds approximately to half the maximum VEGF concentration in the first 24h). Consequently, the VEGF secreted by the cells accumulates inside the construct, hence the increase of VEGF concentration. The long-term decrease is due to the cell density decrease, so that cell secretion of VEGF, despite the oxygen concentration falling below the hypoxic threshold, is no longer enough to compensate for the combined effect of longitudinal diffusion and VEGF degradation.

The VEGF concentration is also spatially non-unform with lower values close to the proximal and distal stumps. This is partly because the concentration of oxygen in the tissue is slightly higher than the hypoxic threshold, causing the cells to secrete less VEGF and partly because of loss to the tissue due to longitudinal diffusion.

Finally, Fig 2 shows that oxygen concentration also decreases non-uniformly. This is mainly due to cell activity, since the time for oxygen metabolism is much smaller (*t*_*c*,M_≈1.95h) axial diffusion time (*t*_*c*,L_≈34.5h≈1.44 days) leading to lower concentration close to the centre of the construct. As for the lower oxygen concentration close to proximal and distal stumps at early times, they are due to loss by diffusion (*c*_0_>*c*_tissue_).

**Fig 3:**
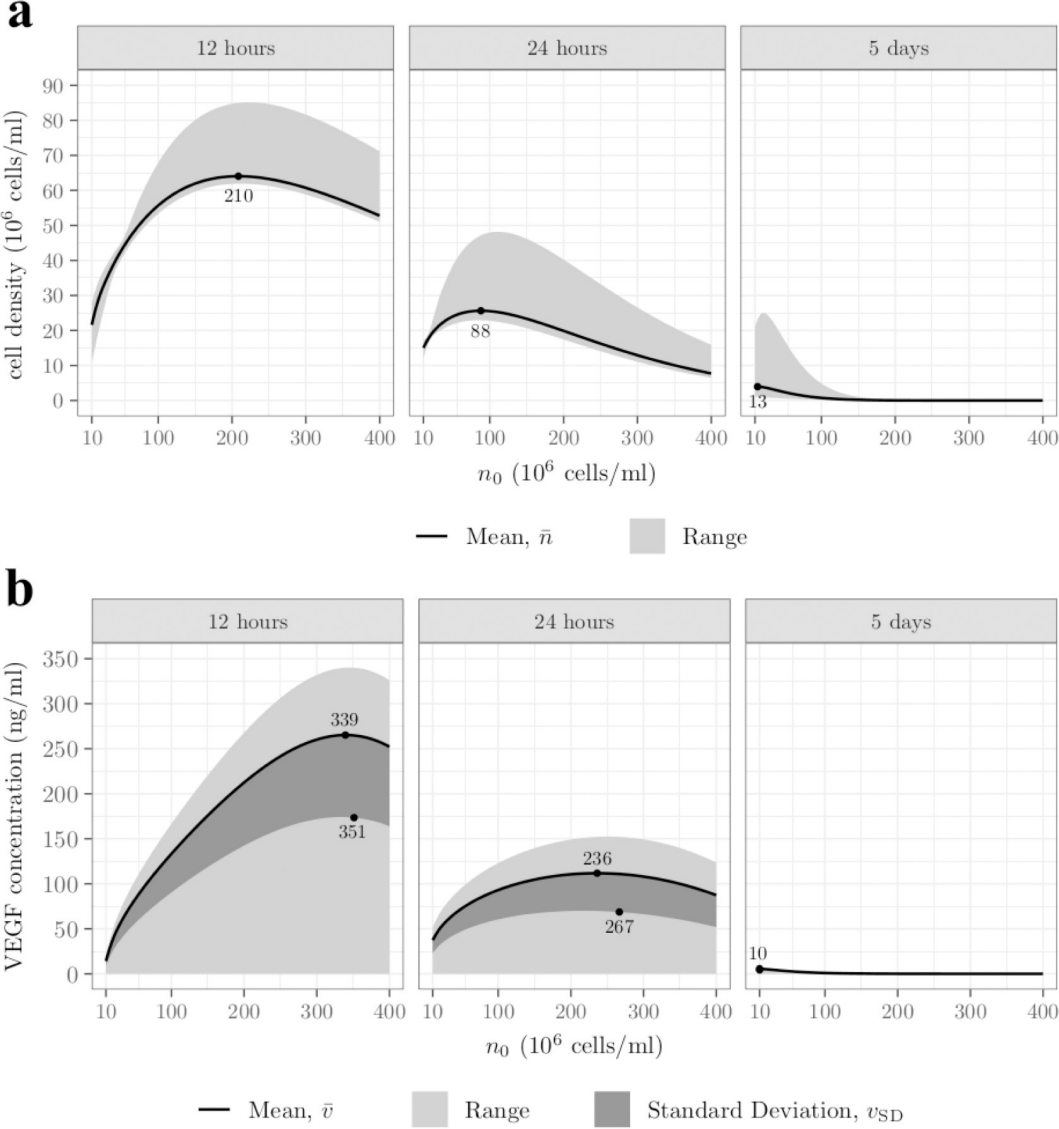
Increasing cell density beyond a threshold decreases both cell survival and VEGF secretion. **A)** cell density and **B)** VEGF concentration means (black lines), standard deviations (dark grey areas) and ranges (light grey areas) as functions of the initial cell density, after 12 hours, 1 day and 5 days. The black dots indicate the initial cell density yielding **A)** the highest mean cell density, *i*.*e*. $\mathrm{argma}x_{n_{0}}\bar{n}$ and **B)** the highest mean VEGF concentration, *i*.*e*. $\mathrm{argma}x_{n_{0}}\bar{v}$, and the highest standard deviation of VEGF concentration, *i*.*e*. $\mathrm{argma}x_{n_{0}}v_{SD}^{}$.

Whereas Fig 2 focuses on a single initial cell density and does not quantitatively inform on the best cell seeding strategy, Fig 3 explores the impact of initial cell density on cell survival and VEGF concentration after 12h, 24h and 120h (5 days). Specifically, Fig 3A shows the mean (black line) and the range (light grey area) associated with cell density as a function of the initial cell density. The black dots represent the initial cell density value which yielded the largest mean cell density at a given time point, *i*.*e*., $\mathrm{argma}x_{n_{0}}\bar{n}$.

After 12h, we see that $\mathrm{argma}x_{n_{0}}\bar{n}=2.10\times 10^{8}$ cells/ml, which corresponds to approximately half the maximal initial cell density tested. Consequently, the model predicts that increasing the initial cell density beyond this value deteriorates cell survival. A larger initial cell density implies a faster consumption of the oxygen initially present in the construct (*e*.*g*., *n*_0_ = 4.00×10^8^ cells/ml yields *t*_*c*,M_ = 0.96h). As a consequence, the rate of change of the cell density becomes negative earlier, causing the cell density to decrease sooner. In addition, the cell death rate *δ* is increasing with *n*_0_, causing the high cell density to decrease faster. On the other hand, a lower initial cell density implies a lower cell death rate along with a slower consumption of the oxygen (*e*.*g*., *n*_0_ = 1.00×10^7^ cells/ml yields *t*_*c*,M_ = 38h) and therefore a longer cell proliferation time. We see, however, that this does not necessarily compensate for the impact of the initial gap in cell density, even though seeding *n*_0_≤4.00×10^7^cells/ml leads to an increase in the mean cell density after 12h. This balance between initial cell density, cell proliferation and death rate explain the maximum value in the mean cell density after 12h.

Fig 3A also shows that, after 12h, the mean cell density is generally much closer to the lower bound of the cell density range, except for very low initial cell density. This is a direct effect of the non-uniformities discussed in Fig 2. For high initial cell densities, the initial oxygen is consumed quickly and oxygen from the tissue cannot penetrate far enough in the construct due to slow axial diffusion and cell consumption close to the stumps. It is possible to estimate the characteristic penetration length of oxygen

$$l_{c}={\left(\frac{c_{tissue}D_{c,g}}{Mn_{max}}\right)}^{\frac{1}{2}},$$ (36)

where *n*_max_ refers to the upper bound of the cell density range which represents, for high initial cell density, the cell density close to the stumps. Such a length is approximately equal to 1.3mm for *n*_max_ = 8.50×10^7^cell/ml (*i*.*e*. the upper bound associated to $argmax_{n_{0}}$ after 12h), so that the region perfused with oxygen (*z*<*l*_*c*_;*z*>*L*−*l*_*c*_) is significantly smaller than the region deprived of oxygen (*l*_*c*_<*z*<*L*−*l*_*c*_), explaining why the mean cell density is closer to the lower bound of the cell density range.

By comparison, for a lower initial cell density, the consumption of oxygen is slower, so that the cell density remains higher close to the centre, resulting in the mean cell density being closer to the upper bound of the range.

Finally, Fig 3A shows that after 12h, the cell density range is minimal for *n*_0_≈4.80×10^7^ cells/ml and maximal for *n*_0_≈2.15×10^8^ cells/ml. This minimal size implies almost uniform cell density. This happens when the region close to the centre starts exhibiting lower cell density than the extremities due to oxygen deprivation. The maximal cell density range, similar to the maximum in mean cell density, is the result of the balance between effects of initial cell density, cell proliferation and cell death rate.

After 24h, Fig 3A shows that the mean cell density has generally decreased but still exhibits the same variations, with a maximum for $\mathrm{argma}x_{n_{0}}\bar{n}=8.8\times 10^{7}$ cells/ml. This decrease is more pronounced for higher initial cell densities due to the continuing lack of oxygen at the centre of the construct and the cell death rate *δ* being an increasing function of *n*_0_. By comparison, lower initial cell densities consume oxygen slower and exhibit lower cell death rates, leading to a smaller decrease over 24h, closing the gap in initial cell density, explaining the decrease of $\mathrm{argma}x_{n_{0}}\bar{n}$.

Fig 3A also shows that after 24h the boundaries of the range have globally decreased except for the lower bound, which has increased for very low initial cell densities (*n*_0_≤2.0×10^7^ cells/ml). This increase being located before the range reaches its minimum size means that cells close to the stumps are still actually proliferating after 24h. This is because the cells in these regions still have access to oxygen from the tissue and are at sufficiently low density so that they don’t have to compete for it.

After 5 days, Fig 3A shows a major change in the cell density behaviour. The cell density reaches zero for *n*_0_>2.00×10^8^ cells/ml, with no cells left alive, even close to the stumps. This is a consequence of the parameter values used to solve Eqs 1–3. To show this, we first determine the stationary states associated with the cell density, which are defined as

$$G_{n}(n_{\mathrm{stat}},c_{g,\mathrm{stat}})-Q_{n}(n_{\mathrm{stat}})=0,$$ (37)

where *n*_stat_ and *c*_g,stat_ denote the stationary cell density and oxygen concentration respectively. Using the expressions for *G*_*n*_ and *Q*_*n*_ (Eqs 2 and 3) the above equation has two solutions

$$n_{\mathrm{stat},1}=0,$$ (38)

$$n_{\mathrm{stat},2}=n_{\max}\left(1-\frac{\delta }{\beta c_{stat}}\right).$$ (39)

Performing a stability analysis of the above two solutions [73] yields

$$\underset{t\to \infty }{\lim}n=n_{\mathrm{stat},1}\;if\;c_{\mathrm{stat}}\leq \frac{\delta }{\beta },$$ (40)

$$\underset{t\to \infty }{\lim}n=n_{\mathrm{stat},2}\;\mathrm{if}\;c_{\mathrm{stat}}>\frac{\delta }{\beta },$$ (41)

which means that the cell density will converge towards zero if the oxygen concentration consistently falls below the survival threshold

$$c_{\tau }=\frac{\delta }{\beta }.$$ (42)

Using the parameter values reported in Table 3 and the range of initial cell density reported in Table 6 (*δ* being an increasing function of *n*_0_) we find that this threshold ranges from *c*_*τ*_ = 4.11%O_2_ for *n*_0_ = 1.00×10^7^ cells/ml to *c*_*τ*_ = 16.43%O_2_ for *n*_0_ = 4.00×10^8^ cells/ml. The tissue being the only long-term source of oxygen with *c*_tissue_ = 5%O_2_, the model will then predict no long-term cell survival for *n*_0_>3.53×10^7^ cells/ml. This is in qualitative agreement with Fig 3A where only very low initial cell densities yield substantial cell survival. This behaviour is completely controlled by the parameter values, which were obtained by Coy et al. [12] using *in vitro* data corresponding to the first 24h. Hence, simulations beyond 24h should be considered as extrapolations and used as qualitative arguments.

With regards to the effect of cell seeding on VEGF secretion, Fig 3B displays the mean (black line), the range (light grey area) and the standard deviation (dark grey area) associated to VEGF concentration as a function of the initial cell density. The black dots represent the position of the initial cell density yielding the highest mean VEGF concentration and largest standard deviation at a given time point, *i*.*e*. $\mathrm{argma}x_{n_{0}}\bar{v}$ and $\mathrm{argma}x_{n_{0}}v_{SD}^{}$. In general, *v*_SD_ represents an indirect measure of VEGF gradient in all directions. However, for the particular case of long and thin ($\frac{R_{1}}{L}\ll 1$) cylinder surrounded by an impermeable sheath, radial gradients have only a small influence so that *v*_SD_ mainly represents longitudinal gradients of VEGF. After 12h, the mean VEGF concentration globally exhibits variations similar to the mean cell density, with a maximum value for $\mathrm{argma}x_{n_{0}}\bar{v}=3.39\times 10^{8}$ cells/ml, meaning that the highest initial cell density does not yield the largest VEGF secretion. High initial cell densities are associated with fast oxygen consumption, resulting in the cell secreting more VEGF at early time points. However, they are also associated with higher cell death (Fig 3A). In addition, effect of VEGF degradation, with characteristic time

$$T_{v,K}=\frac{1}{K},$$ (43)

cannot be neglected (*T*_*v*,*K*_≈9.15h), implying that a VEGF molecule secreted early has likely been degraded after 12h, which is also detrimental to high initial cell density. For lower initial cell densities, on the other hand, the initial oxygen is consumed slower, so that the secretion of VEGF is delayed, hence the lower concentration after 12h. The balance between promoting VEGF secretion via quick oxygen consumption and maintaining a reasonable cell density to sustain meaningful VEGF secretion justifies the existence of a maximum in the mean VEGF concentration after 12h.

Fig 3B also shows that the lower bound of the VEGF concentration range is vanishingly small, which is a direct effect of the boundary condition (Eq 20). On the other hand, the upper bound globally exhibits the same variations as the mean VEGF concentration with a maximum for *n*_0_≈3.44×10^8^cells/ml. This is expected since it represents the concentration of VEGF at the center of the construct, which behaves similarly to the mean VEGF concentration. The mean VEGF concentration is also generally closer to the upper bound of the range. The region perfused by oxygen from the tissue is relatively small (Eq 36) so that cell in the large central region are deprived of oxygen and secrete more VEGF. Given the slow axial diffusion, the concentration in VEGF in this region increases, and since it has a large volume, it contributes more to the mean VEGF concentration, which ends up being closer to the upper bound of the range.

Fig 3A finally shows that the standard deviation also globally follows the same variations as the mean and the upper bound of the VEGF concentration, with a maximum for $\mathrm{argma}x_{n_{0}}v_{SD}^{}=3.49\times 10^{8}$ cells/ml. We recall that the standard deviation serves as a global measure of the longitudinal VEGF concentration gradient, *i*.*e*., the difference between VEGF concentration at the centre (upper bound) and the extremities (lower bound). The latter being constant regardless of the initial cell density, the standard deviation is actually controlled by the upper bound, which explains its behaviour. The difference between $\mathrm{argma}x_{n_{0}}v_{SD}^{}$ and $\mathrm{argma}x_{n_{0}}\bar{v}$ is due to the integration of axial non-uniformities in the VEGF concentration.

After 24h, Fig 3B shows that the mean VEGF concentration still exhibits the same variations with a maximum for $\mathrm{argma}x_{n_{0}}\bar{v}=2.36\times 10^{8}$ cells/ml but has generally decreased except for *n*_0_≤2.5×10^7^. Low initial cell densities are associated with slower oxygen consumption, sustaining longer cell proliferation (Fig 3A). This causes the VEGF secretion to peak later, explaining the increase in mean VEGF concentration. On the contrary, high initial cell densities are associated with an earlier peak in VEGF secretion. A large fraction of VEGF molecules secreted at that time have then likely been degraded after 24h, implying that the remaining concentration is the result of the much lower cell density (Fig 3A), explaining the decrease in the mean VEGF concentration. This balance between cell density, VEGF degradation and the peak in VEGF secretion also explains $\mathrm{argma}x_{n_{0}}\bar{v}$ decrease.

Fig 3B also shows that the standard deviation exhibits the same behaviour as the mean VEGF concentration with $\mathrm{argma}x_{n_{0}}v_{SD}^{}=n_{0}\approx 2.67\times 10^{8}$ cells/ml. We note that the difference between $\mathrm{argma}x_{n_{0}}v_{SD}^{}$ and $\mathrm{argma}x_{n_{0}}\bar{v}$ has increased, which is the consequence of the continuing time integration of longitudinal non-uniformities in the VEGF concentration.

After 5 days, the VEGF mean, range and standard deviation have collapsed towards zero for most initial cell densities. At this time, only cells located close to the stumps remain alive. There, the oxygen level is above the hypoxic threshold so that the VEGF secretion is hindered. The combined effect of degradation and axial diffusion, which is no longer negligible (*t*_*v*,L_≈5.8 days), having eliminated the pre-existing VEGF concentration, explains the vanishingly small concentration.

#### Culture conditions

**Fig 4:**
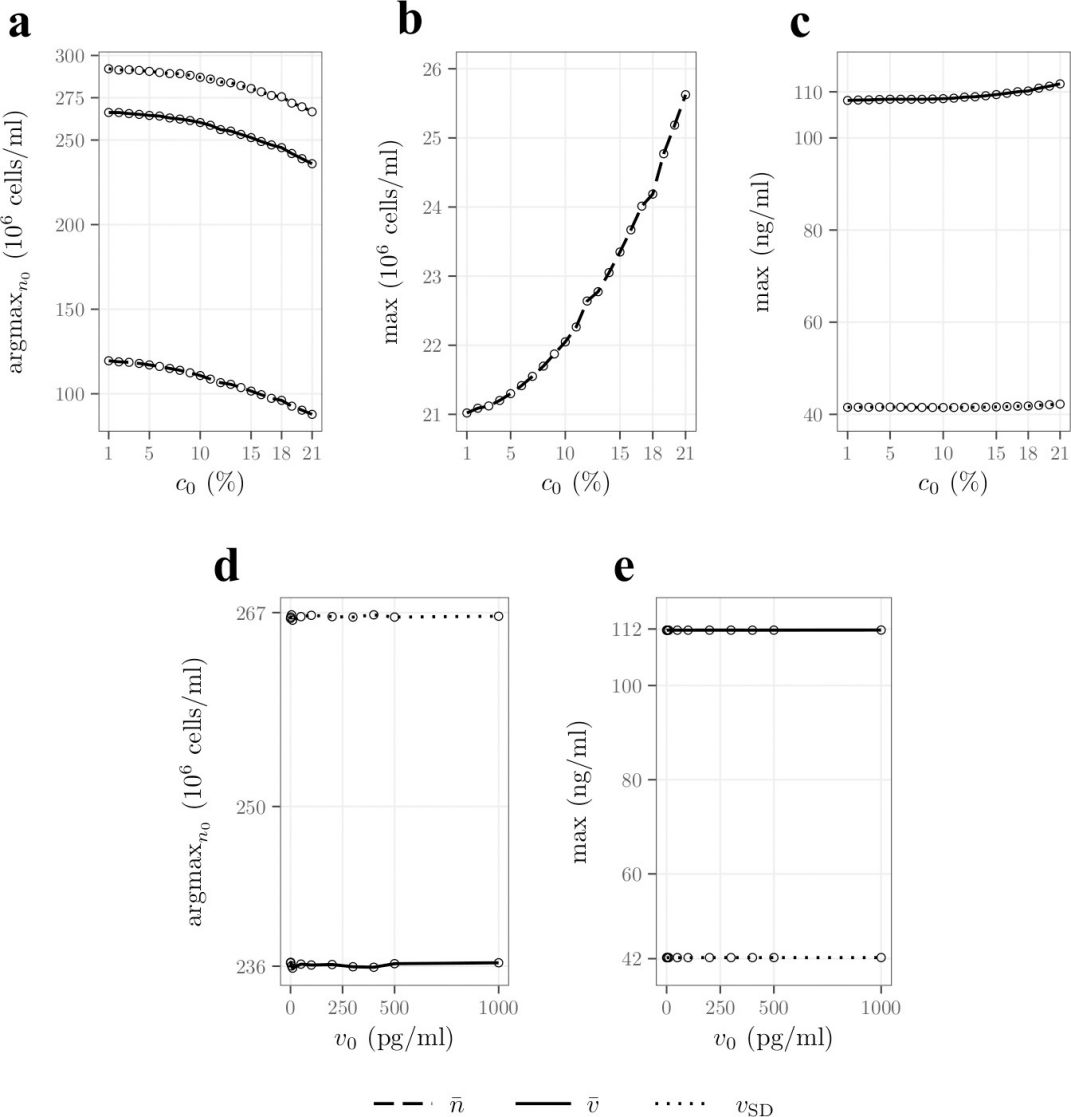
Increasing the initial oxygen concentration supports fewer seeded cells and results in an increased cell density and VEGF concentration and gradient after 24h. **A)** initial cell density yielding the highest cell density ($\mathrm{argma}x_{n_{0}}\bar{n}$, dashed line), the highest VEGF concentration ($\mathrm{argma}x_{n_{0}}\bar{v}$, continuous line) and the largest standard deviation in the VEGF concentration ($\mathrm{argma}x_{n_{0}}v_{SD}^{}$, dotted line) as functions of the initial concentration of oxygen *i*.*e*. *c*_0_. **B)** $\max_{n_{0}}\bar{n}$ as a function of *c*_0_. **C)** $\max_{n_{0}}\bar{v}$ (continuous line) and $\max_{n_{0}}v_{SD}$ (dotted line) as functions of *c*_0_. **D)** $\mathrm{argma}x_{n_{0}}\bar{v}$ (continuous line) and $\mathrm{argma}x_{n_{0}}v_{SD}^{}$ (dotted line) as functions of the initial concentration of VEGF *i*.*e*. *v*_0_. **E)** $\max_{n_{0}}\bar{v}$ (continuous line) and $\max_{n_{0}}v_{SD}$ (dotted line) as functions of *v*_0_.

In this section we quantify the impact of the initial oxygen (*c*_0_) and VEGF (*v*_0_) concentrations on cell survival and VEGF secretion. These conditions can be controlled experimentally to improve outcomes post-implantation. Fig 4A shows the initial cell density yielding the highest cell density ($\mathrm{argma}x_{n_{0}}\bar{n}$, dashed line), the highest VEGF concentration ($\mathrm{argma}x_{n_{0}}\bar{v}$, plain line) and the highest standard deviation in VEGF concentration ($\mathrm{argma}x_{n_{0}}v_{SD}^{}$, dotted line) as functions of *c*_0_ after 24h. All three functions are decreasing, implying that lower initial cell densities yield better performances for high initial oxygen concentration. This is expected for $\mathrm{argma}x_{n_{0}}\bar{n}$ since a higher initial oxygen concentration implies a longer consumption time (Eq 33) and therefore longer proliferation times. This situation favours lower initial cell densities after 24h (Fig 3A), explaining the behaviour of $\mathrm{argma}x_{n_{0}}\bar{n}$. These longer oxygen consumption and proliferation times also result in a delay in the peak of VEGF secretion, which also favours lower initial cell densities after 24h (Fig 3B), explaining the behaviour of $\mathrm{argma}x_{n_{0}}\bar{v}$ and $\mathrm{argma}x_{n_{0}}v_{SD}^{}$.

We note that $\mathrm{argma}x_{n_{0}}\bar{v}\neq \mathrm{argma}x_{n_{0}}v_{SD}^{}$ so that there is no initial cell density that maximises both the mean VEGF concentration and the VEGF gradient. The differences between these two quantities are almost constant regardless of the initial oxygen concentration and is the result of the longitudinal non-uniformities in the VEGF concentration, which are not sensitive to the initial concentration of oxygen.

Fig 4B shows the maximum mean cell density, *i*.*e*., $\mathrm{ma}x_{n_{0}}\bar{n}$, as a function of the initial oxygen concentration in the construct. We can see that it increases with *c*_0_, which is expected since increasing the initial oxygen concentration is associated with an increase in the cell proliferation time resulting in an increase in the cell density after 24h.

Similarly, Fig 4C shows that the maximum mean VEGF concentration and maximum VEGF standard deviation, *i*.*e*., $\mathrm{ma}x_{n_{0}}\bar{v}$ and $\mathrm{ma}x_{n_{0}}v_{SD}$, are also slightly increasing functions of *c*_0_. This is consistent with the increase in $\mathrm{ma}x_{n_{0}}\bar{n}$ as a higher cell density, under low oxygen conditions, secretes more VEGF.

Finally, Fig 4D shows $\mathrm{argma}x_{n_{0}}\bar{v}$ and $\mathrm{argma}x_{n_{0}}v_{SD}^{}$ and Fig 4E shows $\mathrm{ma}x_{n_{0}}\bar{v}$ and $\mathrm{ma}x_{n_{0}}v_{SD}^{}$, as functions of the initial VEGF concentration in the construct *v*_0_. We can see that *v*_0_ yields little to no effects on these quantities. This can be explained since most of the VEGF initially present has been degraded after 24h (Eq 43). In addition, $\mathrm{ma}x_{n_{0}}\bar{v}\approx 112$ ng/ml which is much higher than *v*_0_, explaining the constant behaviour of $\mathrm{argma}x_{n_{0}}\bar{v}$. The same reasoning applies for $\mathrm{ma}x_{n_{0}}v_{SD}^{}$ and $\mathrm{argma}x_{n_{0}}v_{SD}^{}$ recalling that *v*_SD_ is mainly controlled by the concentration at the centre of the construct.

#### Surrounding tissue

**Fig 5:**
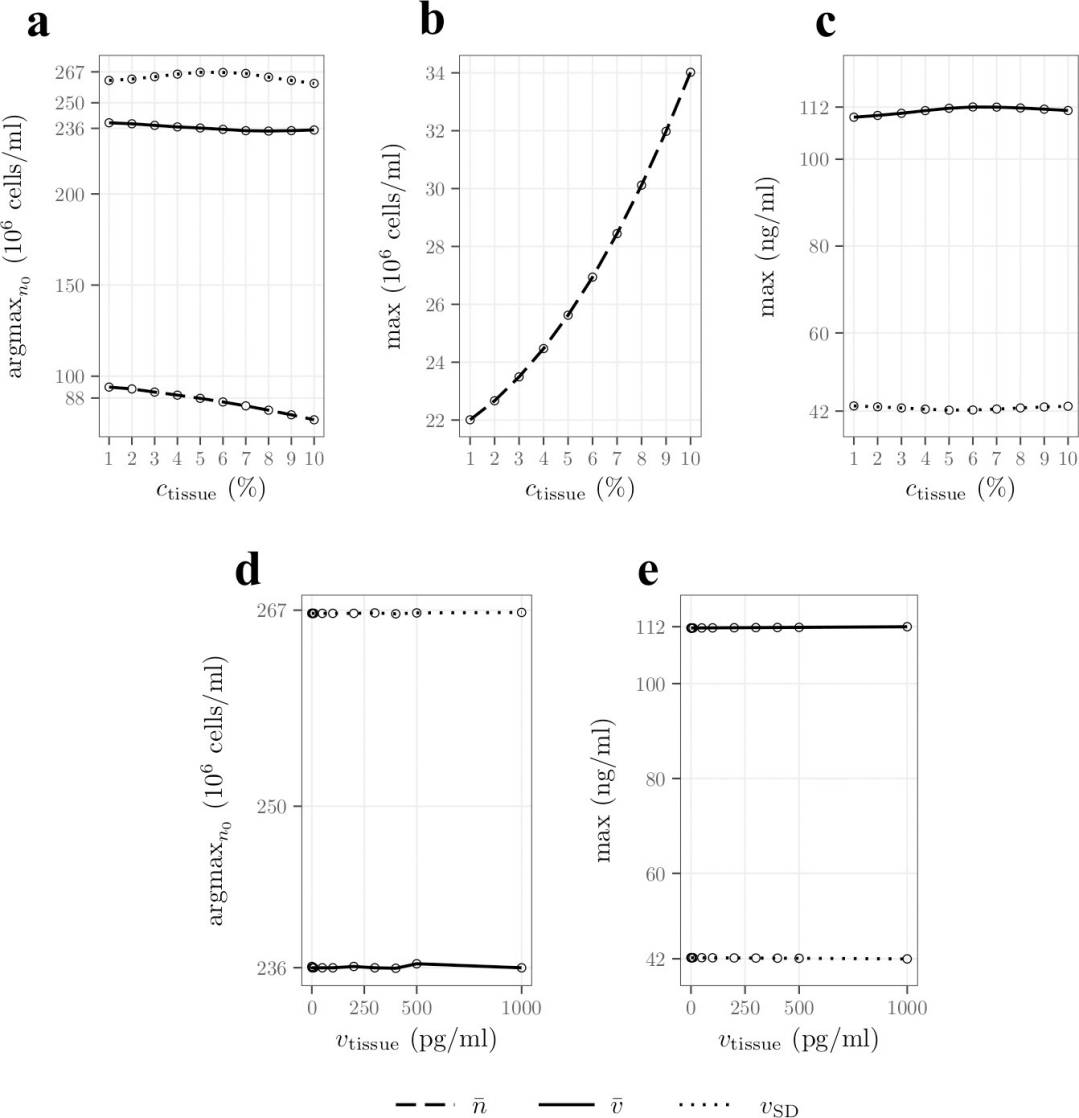
Increasing the surrounding oxygen tissue concentration supports fewer seeded cells and results in an increased cell density after 24h. **A)** initial cell density yielding the highest cell density ($\mathrm{argma}x_{n_{0}}\bar{n}$, dashed line), the highest VEGF concentration ($\mathrm{argma}x_{n_{0}}\bar{v}$, continuous line) and the largest VEGF concentration standard deviation ($\mathrm{argma}x_{n_{0}}v_{SD}^{}$, dotted line) as functions of the concentration of oxygen in the surrounding tissue, *i*.*e*., *c*_tissue_. **B)** $\max_{n_{0}}\bar{n}$ as a function of *c*_tissue_. **C)** $\max_{n_{0}}\bar{v}$ (continuous line) and $\max_{n_{0}}v_{SD}$ (dotted line) as functions of *c*_tissue_. **D)** $\mathrm{argma}x_{n_{0}}\bar{v}$ (continuous line) and $\mathrm{argma}x_{n_{0}}v_{SD}^{}$ (dotted line) as functions of the concentration of VEGF in the surrounding tissue *i*.*e*., *v*_tissue_ on a logscale. **E)** $\max_{n_{0}}\bar{v}$ (continuous line) and $\max_{n_{0}}v_{SD}$ (dotted line) as functions of *v*_tissue_ on a logscale.

In this section we investigate the impact of the tissue oxygen concentration (*c*_tissue_) and tissue VEGF concentration (*v*_tissue_) upon cell survival and VEGF concentration. Both of these parameters will depend on the specific repair scenario and are challenging to define due to a paucity of direct measurements in the literature. Therefore, it is important to understand the impact of these parameters on model predictions. Fig 5A shows the initial cell density yielding the highest cell density ($\mathrm{argma}x_{n_{0}}\bar{n}$, dashed line), the highest VEGF concentration ($\mathrm{argma}x_{n_{0}}\bar{v}$, plain line) and the largest standard deviation in the VEGF concentration ($\mathrm{argma}x_{n_{0}}v_{SD}^{}$, dotted line) as functions of *c*_tissue_ after 24h. Similar to Fig 4A, $\mathrm{argma}x_{n_{0}}\bar{n}$ is decreasing, so that lower initial cell densities perform best with a high tissue oxygen concentration. This can be explained since an increase in oxygen concentration, even when predominantly located close to the proximal and distal stumps, favours low initial cell densities (Fig 3A).

On the contrary, $\mathrm{argma}x_{n_{0}}\bar{v}$ and $\mathrm{argma}x_{n_{0}}v_{SD}^{}$ are not sensitive to an increase in the tissue oxygen concentration. An increase in *c*_tissue_ mainly reduces VEGF secretion by cells in the oxygen-perfused regions (Eq 36). In these regions, the VEGF concentration is already low, due to the combined effect of the tissue VEGF concentration (*v*_tissue_ = 0) and longitudinal diffusion. Consequently, $\bar{v}$ and *v*_SD_, which are mainly controlled by the VEGF concentration in the central region of the construct (Fig 3B), remain relatively unaffected by changes in *c*_tissue_.

Analogous to Fig 4B, Fig 5B shows that $\mathrm{ma}x_{n_{0}}\bar{n}$ is an increasing function of *c*_tissue_, as cells close to proximal and distal stumps have access to a higher oxygen concentration and therefore proliferate more.

On the other hand, Fig 5C shows that $\mathrm{ma}x_{n_{0}}\bar{v}$ and $\mathrm{ma}x_{n_{0}}v_{SD}$, are not sensitive to an increase in the tissue oxygen concentration. $\bar{v}$ and *v*_SD_, $\mathrm{ma}x_{n_{0}}\bar{v}$ and $\mathrm{ma}x_{n_{0}}v_{SD}$, are mainly controlled by the VEGF concentration in the central region of the construct, which is not significantly affected by an increase in *c*_tissue_.

Finally, Fig 5D and 5E show $\mathrm{argma}x_{n_{0}}\bar{v},\;\mathrm{argma}x_{n_{0}}v_{SD}^{}$ and $\mathrm{ma}x_{n_{0}}\bar{v},\;\mathrm{ma}x_{n_{0}}v_{SD}^{}$ respectively, as functions of the tissue concentration of VEGF. Similar to Fig 4D and 4E, none of them is sensitive to *v*_tissue_. This is because $\mathrm{ma}x_{n_{0}}\bar{v}$ and $\mathrm{ma}x_{n_{0}}v_{SD}^{}$ are much higher than *v*_tissue_ meaning that they are completely controlled by the VEGF secreted in the construct, also explaining the constant behaviour of $\mathrm{argma}x_{n_{0}}\bar{v}$ and $\mathrm{argma}x_{n_{0}}v_{SD}$.

### Porous sheath

In this section, we investigate the effect of sheath thickness, porosity and tortuosity upon cell survival, oxygen concentration and VEGF concentration. The goal is to use insights from the model to inform which sheath properties enable improved NRC performance after 1 day.

**Fig 6:**
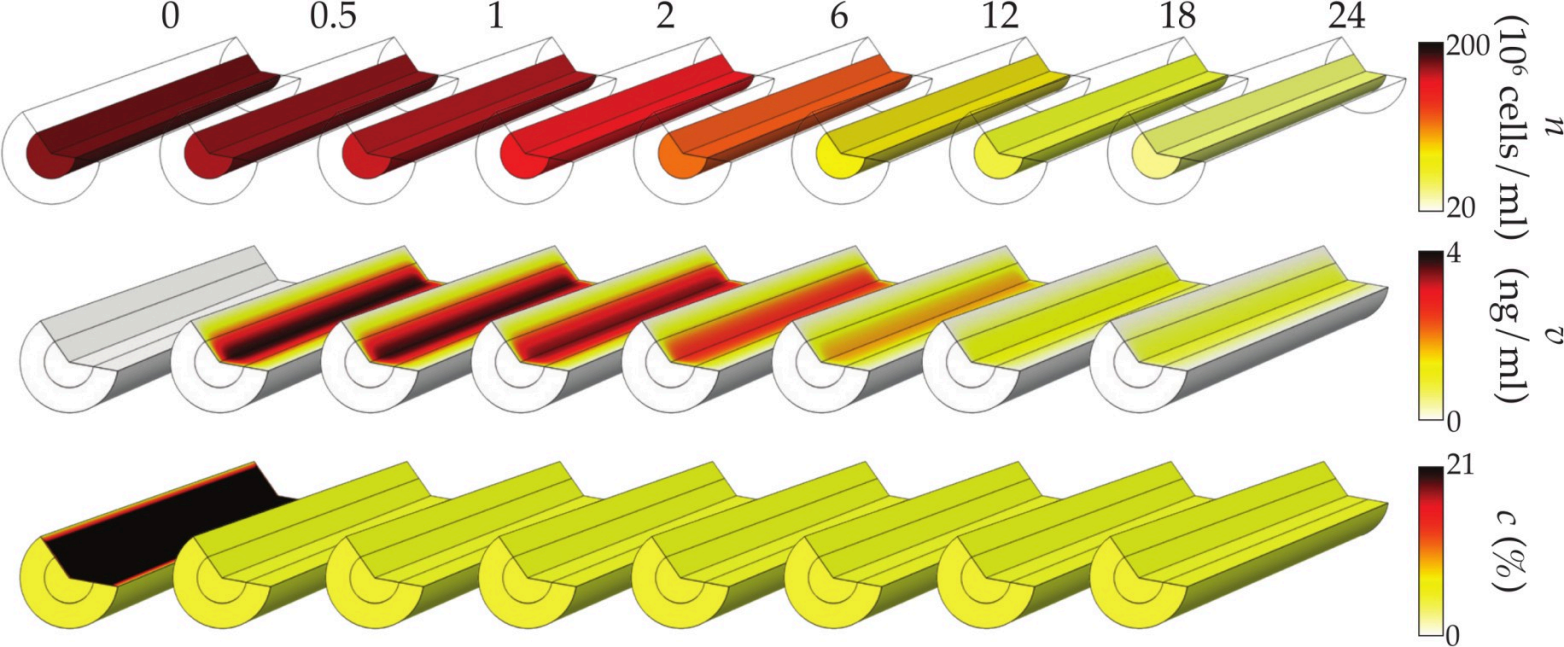
Cell-solute interactions in a permeable nerve repair construct results in a quick decrease in oxygen concentration and a low uniform VEGF concentration. Heatmaps of cell density, oxygen and VEGF concentration over 24 hours for an initial cell density *n*_0_ = 1.78×10^6^ cells/ml and a porosity *ϵ* = 0.8. The porous sheath structure is approximated by an array of overlapping spheres (see Table 1) with a thickness *T* = 0.25mm and a porosity *ϵ* = 0.8.


Fig 6 shows heatmaps of cell density, VEGF and oxygen concentrations over 24 hours. The cell density decreases over time despite cells having better access to oxygen, although this decrease is smaller than for the impermeable scenario. This decrease is due to the large cell death rate, which is not compensated by the improved access to oxygen. Contrarily to an impermeable sheath however, there are no discernible longitudinal heterogeneities in the cell density as *c*_tissue_ is uniform so cells have similar access to oxygen regardless of their axial position. This holds only because the sheath has a large porosity (*ϵ* = 0.8) and a relatively small thickness (*T* = 0.25mm) so the radial diffusion time of oxygen in the construct

$$t_{c,R}=\frac{R_{1}^{2}}{D_{c,g}}+\frac{\tau^{2}T^{2}}{D_{c,s}},$$ (44)

is much shorter (*t*_*c*,R_≈0.05h) than its axial counterpart (*t*_*c*,L_≈34.5h≈1.44 days).

The VEGF concentration evolves similarly, although much faster, to the case of an impermeable sheath (Fig 2). The concentration is much lower with no discernible axial gradients. This is due to the combined effect of zero tissue VEGF concentration and the radial diffusion time of VEGF in the construct

$$t_{v,R}=\frac{R_{1}^{2}}{D_{v,g}}+\frac{\tau^{2}T^{2}}{D_{v,s}},$$ (45)

being much shorter (*t*_*v*,R_≈0.31h) than its longitudinal counterpart (*t*_*v*,L_≈139h≈5.8 days), and the secretion time of VEGF (*t*_*v*,P_≈3.63h). This equilibrium between radial diffusion and VEGF secretion explains why VEGF concentration rises to such low values at early times, and the difference between radial and longitudinal diffusion times explains why there are no longitudinal gradients. The long-term decrease of VEGF is due to the cell density decrease combined with the oxygen concentration in the tissue being higher than the hypoxic threshold (*c*_tissue_>*c*_*h*_).

Finally, Fig 6 shows that oxygen concentration relaxes from *c*_0_ to *c*_tissue_ in under 0.5h. Such a quick relaxation is due to the combination of the cell density being the highest at early times, which implies stronger oxygen consumption, with the radial diffusion time of oxygen in the construct being small (*t*_*v*,R_≈0.05h).

**Fig 7:**
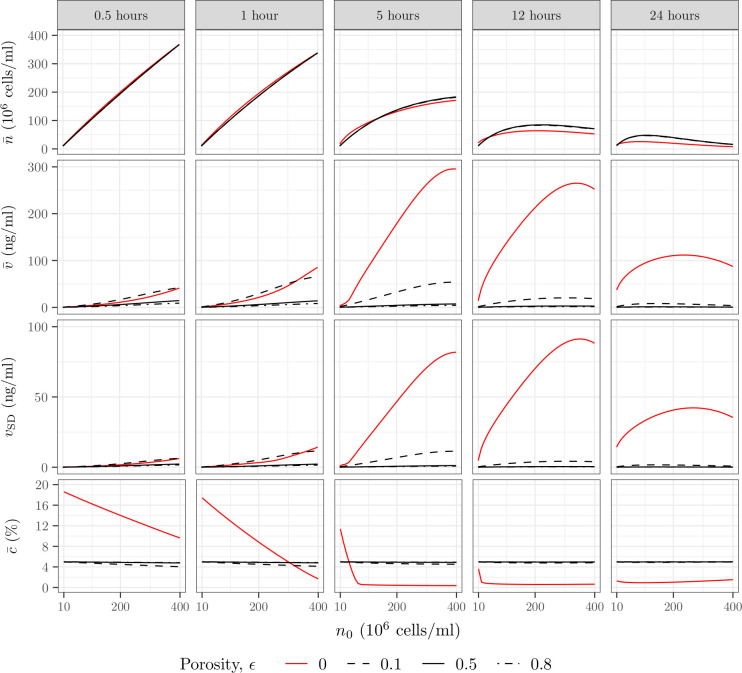
Increasing the sheath porosity of a permeable construct results in a higher cell density and oxygen concentration and a much lower VEGF concentration and gradient after 24h. Mean cell density $\bar{n}$, mean oxygen concentration $\bar{c}$, mean VEGF concentration $\bar{v}$ and standard deviation *v*_SD_, as functions of the initial cell density *n*_0_, for different sheath porosity *ϵ*, at different time points. The porous sheath structure is approximated by an array of overlapping sphere (see Table 1) with a thickness *T* = 0.25mm. We note that *ϵ* = 0 corresponds to an impermeable sheath (red lines).

Fig 6 focuses on a single initial cell density and a single porosity and does not quantitatively inform the best cell seeding strategy. By comparison, Fig 7 explores the impact of seeded cell density for different sheath porosities. Consistent with Fig 3A, the oxygen concentration decreases faster for higher initial cell densities when the sheath is impermeable. We also note that when the sheath is porous the concentration in oxygen remains globally constant regardless of the initial cell density and is close to *c*_tissue_ in under 0.5h, even for *ϵ* = 0.1, which is associated with a smaller radial diffusive flux (Eq 15). This is a direct consequence of the radial diffusion time of oxygen (*t*_*c*,*R*_<0.1h) being much smaller than the consumption time of oxygen (0.96h<*t*_*c*,*M*_<38h), so that the radial diffusive flux is not limiting.

With regard to cell survival, we see that for *t*<1h the cell density associated with the impermeable sheath is slightly higher than the cell densities associated with a porous sheath, regardless of *n*_0_. This is due to the quick loss of oxygen by radial diffusion when the sheath is porous. Even after 12h, for *n*_0_≤4×10^7^cells/ml, the impermeable sheath still performs slightly better than the porous sheath despite exhibiting lower oxygen concentration. That is because the oxygen concentration only affects the cell proliferation, *i*.*e*. the derivative of the cell density, and not the cell density directly. This introduces a delay between the moment when the oxygen concentration in the impermeable case becomes smaller than in the porous case, and the moment when the cell density effectively becomes lower than in the porous case. After 24h, the oxygen concentration in the impermeable case has been lower long enough so that the associated mean cell density is also lower than the porous case, regardless of *n*_0_.

After 12h and 24h both $max_{n_{0}}\bar{n}$ and $argmax_{n_{0}}\bar{n}$ are higher when the sheath is porous ($argmax_{n_{0}}\bar{n}\approx 1.10\times 10^{8}$ cells/ml and $max_{n_{0}}\bar{n}\approx 5.00\times 10^{7}$ cells/ml after 24h for *ϵ*≥0.1) as the cells have access to oxygen supplied by the tissue. Finally, we note that the cell density has the same behaviour regardless of the porosity, which aligns with the mean oxygen concentration also being insensitive to porosity.

With regards to VEGF secretion, a porous sheath dramatically decreases VEGF concentration after 24h compared to an impermeable sheath. However, after 1h both the mean and standard deviation of the VEGF concentration are higher for a porous sheath with *ϵ* = 0.1 than for an impermeable sheath, except for cell densities above *n*_0_>3.30×10^8^cells/ml. This is because 1) the oxygen concentration is lower when the sheath is porous except for *n*_0_>3.30×10^8^cells/ml, so that cells will secrete more VEGF, 2) *ϵ* = 0.1 implies a small radial diffusive flux between the collagen cylinder and the annular sheath (*E*q 16) and 3) the radial diffusion time is long enough (*t*_*v*,*R*_ = 0.63h) so that VEGF concentration can build inside the construct. In line with this observation, increasing the porosity leads to a significant decrease in the mean VEGF concentration and standard deviation.

#### Sheath material

**Fig 8:**
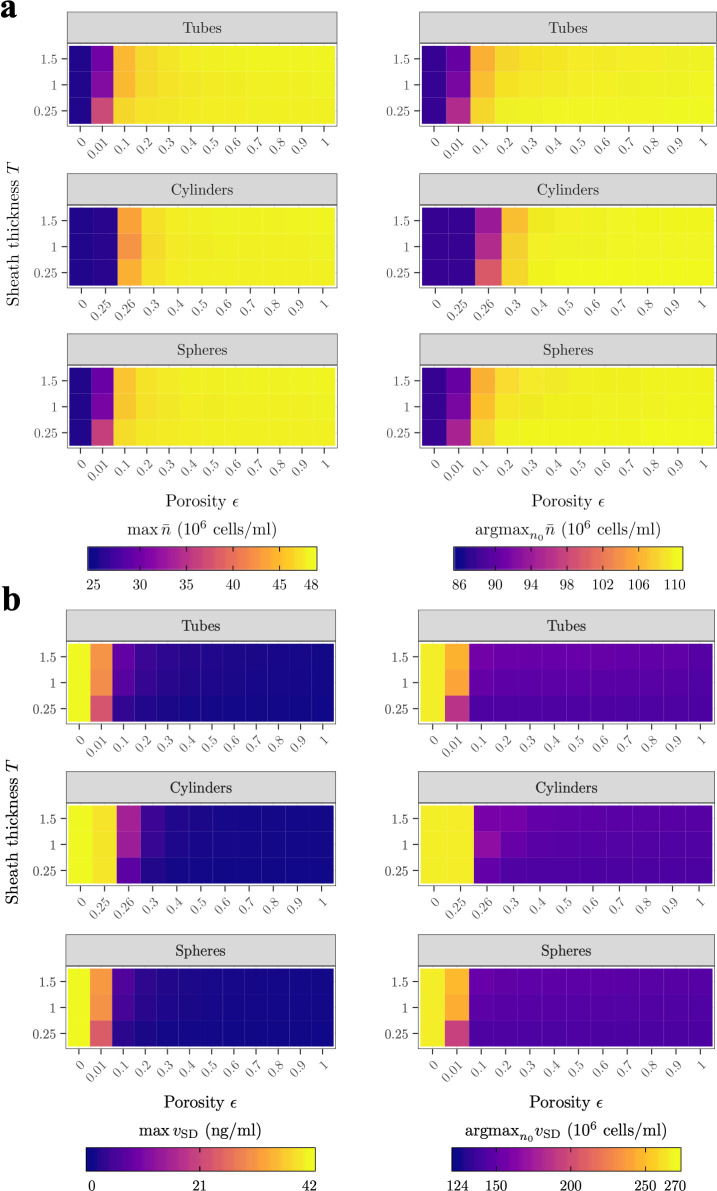
Increasing sheath porosity increases the maximum cell density and decreases the VEGF concentration and gradient field after 24h, regardless of the underlying archetypal porous structure. **Increasing sheath thickness has opposite effects. A)** $\max_{n_{0}}\bar{n}$ and the associated initial cell density $\mathrm{argma}x_{n_{0}}\bar{n}$ and **B)** $\max_{n_{0}}v_{SD}$ and the associated initial cell density $\mathrm{argma}x_{n_{0}}v_{SD}^{}$ as functions of sheath porosity *ϵ* and sheath thickness *T* for the different tortuosity expressions listed in Table 1.

In this section, we investigate the effect of sheath tortuosity, porosity and thickness on cell survival and VEGF secretion. Fig 8A shows, for each porous structure introduced in Table 1, $\mathrm{argma}x_{n_{0}}\bar{n}$ and the associated maximum cell density $\mathrm{ma}x_{n_{0}}\bar{n}$ as functions of the sheath porosity and thickness after 24h. We see that for porosity in the range 0.3≤*ϵ*≤1 all three porous structures yield similar performances. For smaller porosity (*ϵ*<0.3), however, we see that the cylindrical structure differs significantly from the two others. That is because the associated tortuosity model has been asymptotically derived for large porosity and exhibits a singularity at *ϵ* = 0.25. We also note that increasing the porosity results in a sharp increase of $\mathrm{argma}x_{n_{0}}\bar{n}$ and $\mathrm{ma}x_{n_{0}}\bar{n}$ for the spherical and tubular structures that plateaus for *ϵ*≥0.3 because cells have better access to oxygen via radial diffusion. Small porosities are associated with a small radial flux of oxygen ($\frac{\epsilon }{\tau }\ll 1$ in Eq 15) so that increasing *ϵ* relaxes such constraint.

Finally, we see that increasing the sheath thickness decreases $\mathrm{argma}x_{n_{0}}\bar{n}$ and $\mathrm{ma}x_{n_{0}}\bar{n}$ noticeably for *ϵ*≤0.3. An increase in sheath thickness implies an increase in radial diffusion time of oxygen (*e*.*g*. *t*_*c*,R_≈0.05h for *T* = 0.25mm and *t*_*c*,R_≈0.5h for *T* = 1.5mm) so that cells have an impaired access to oxygen, especially for small porosities, which are already associated with a limited flux of oxygen ($\frac{\epsilon }{\tau }\ll 1$ in Eq 15). This explains the decrease in $\mathrm{argma}x_{n_{0}}\bar{n}$ and $\mathrm{ma}x_{n_{0}}\bar{n}$.

Fig 8B shows $\mathrm{argma}x_{n_{0}}v_{SD}$ and $\mathrm{ma}x_{n_{0}}v_{SD}$ as functions of the sheath porosity and thickness after 24h. Results with regard to the mean VEGF concentration were not reported here as they were very similar. Both $\mathrm{argma}x_{n_{0}}v_{SD}$ and $\mathrm{ma}x_{n_{0}}v_{SD}$ are globally not sensitive to the underlying porous structure. Contrarily to cell density, however, increasing sheath porosity decreases $\mathrm{argma}x_{n_{0}}v_{SD}$ and $\mathrm{ma}x_{n_{0}}v_{SD}$. This is due to the combined effect of the relatively short radial diffusion time and the increase in the flux of VEGF at the interface between collagen and sheath.

Finally, increasing sheath thickness also increases $\mathrm{argma}x_{n_{0}}v_{SD}$ and $\mathrm{ma}x_{n_{0}}v_{SD}$, especially for small sheath porosity. This is expected as a thicker sheath implies a longer radial diffusion time for VEGF (*e*.*g*., *t*_*v*,R_≈0.31h for *T* = 0.25mm and *t*_*v*,R_≈5.22h for *T* = 1.5mm, assuming *ϵ* = 0.8 and a spherical porous structure) so that molecules can accumulate in the construct. Increasing sheath thickness also impairs access to oxygen in the main body of the NRC, so that cells rely on the initial oxygen concentration to survive. Higher initial cell densities tend to produce more VEGF due to quick oxygen consumption, hence the increase in $\mathrm{argma}x_{n_{0}}v_{SD}$.

#### Partially porous sheath

In this section, we investigate the impact of a sheath that is porous at the extremities and impermeable at the centre. This is motivated by the idea that a partially porous sheath could serve as a compromise between the impermeable case that favours VEGF secretion, and the porous case that favours cell survival.

**Fig 9:**
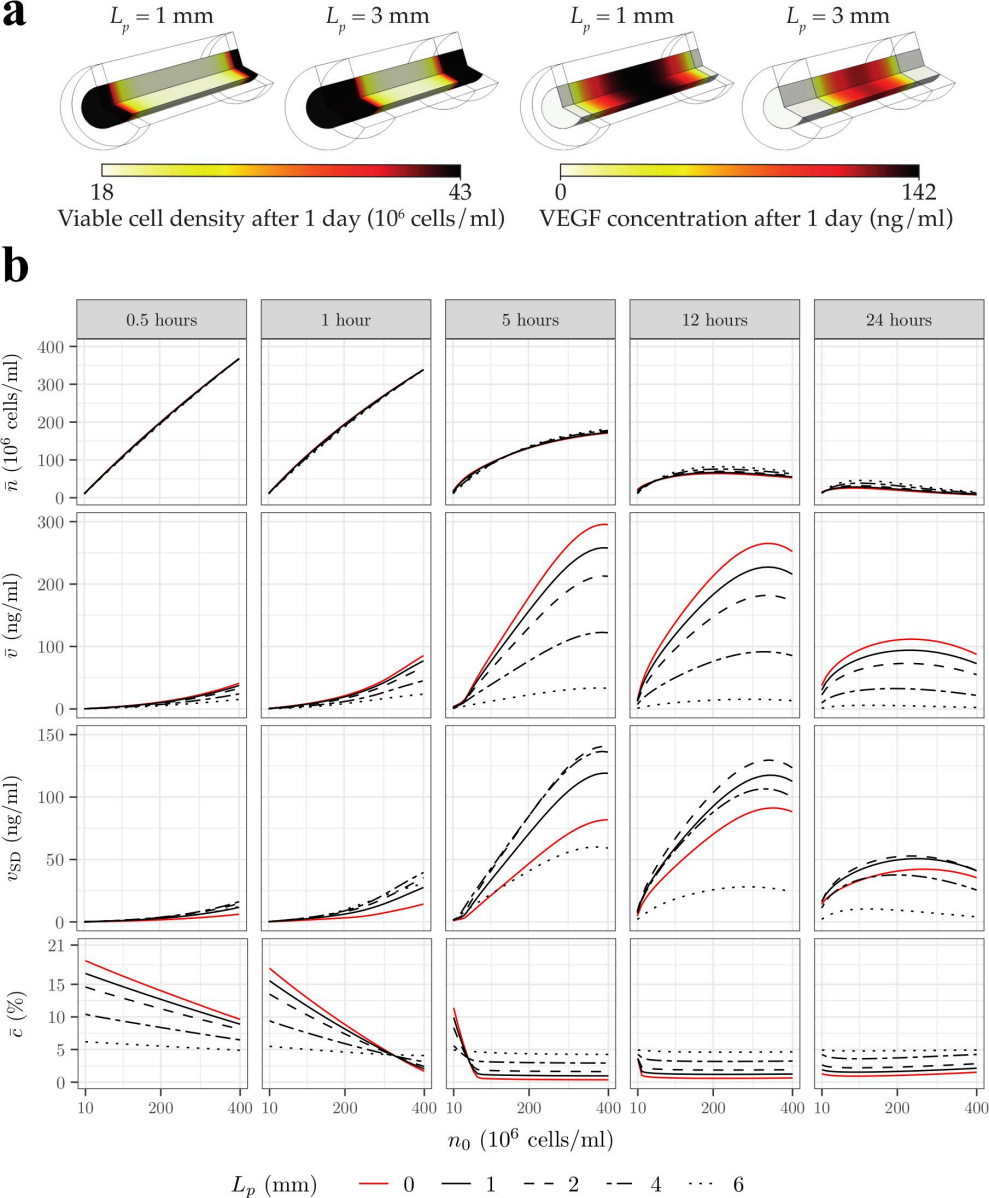
A partially porous sheath with a moderate length *L*_*p*_ limits the decrease of VEGF concentration while still increasing cell density and oxygen concentration after 24h. **A)** Heatmaps of cell density and VEGF concentration after 1 day, for two different porous sheath zone lengths *L*_*p*_, for an initial cell density *n*_0_ = 1.78×10^6^ cells/ml. **B)** Mean cell density $\bar{n}$, mean oxygen concentration $\bar{c}$ and mean VEGF concentration $\bar{v}$ and standard deviation *v*_SD_, as functions of the initial cell density n_0_, for different *L*_*p*_ values over 1 day. In **A)** and **B)** the porous sheath structure is approximated by an array of overlapping spheres (see Table 1) with a thickness *T* = 0.25mm and a porosity *ϵ* = 0.8. We note that L_p_ = 0 (red lines) corresponds to an impermeable sheath.

Fig 9A shows heatmaps of cell density and VEGF concentration after 24h, for two partially porous sheaths (*ϵ* = 0.8 and *T* = 0.25mm) with porous length *L*_*p*_ = 1mm and *L*_*p*_ = 3mm respectively. We can see that the porous regions exhibit higher cell density and lower VEGF concentration compared to the impermeable region, which is consistent with results reported in Fig 7, and is due to short radial diffusion times and the cells having improved access to oxygen. By comparison, the central region has only access to oxygen through longitudinal diffusion, which is still significantly slower (*t*_*c*,L_≈1.1 days for *L*_*p*_ = 1mm and *t*_*c*,L_≈12.5h for *L*_*p*_ = 3mm substituting *L* by *L*−2*L*_*p*_ in Eq 32) than the consumption of oxygen (*e*.*g*. for *n*_0_ = 1.78×10^8^ cells/ml, *t*_*c*,M_≈1.95h), so that cells in this region mostly rely on the initial oxygen supply. This initial oxygen reserve is consumed within a few hours, leading to lower oxygen concentration and lower cell density after 24h. By comparison, the slow longitudinal diffusion timescale for VEGF in the central region (*t*_*v*,L_≈4.3 days for *L*_*p*_ = 1mm and *t*_*v*,L_≈2 days for *L*_*p*_ = 3mm substituting *L* by *L*−2*L*_*p*_ in Eq 34) together with the low oxygen concentration there gives rise to a higher VEGF concentration.

We still note that increasing *L*_*p*_ results in an increase in cell density and a decrease in VEGF concentration in the central region, mainly due to the reduction in oxygen and VEGF longitudinal diffusion times.

Fig 9B extends these results and shows the mean cell density, VEGF and oxygen concentration, as well as the standard deviation in the VEGF concentration, as functions of the initial cell density for different length of the porous sheath zones (*L*_*p*_). After 0.5h, the mean oxygen concentration decreases with *L*_*p*_ regardless the initial cell density. This is because 1) oxygen concentration in the porous regions quickly decreases to reach *c*_tissue_ and 2) increasing *L*_*p*_ decreases the longitudinal diffusion time in the impermeable region (substituting *L* by *L*−2*L*_*p*_ in Eq 32), resulting in the observed decrease in mean oxygen concentration. After 1h, however, we see that the mean oxygen concentration starts to increase with the porous length for high initial cell densities (*n*_0_>3.30×10^8^cells/ml). This is a consequence of the short oxygen consumption time, which causes the oxygen concentration in the impermeable region to decrease below the concentration in the porous region. Increasing *L*_*p*_ hence increases the mean oxygen concentration by reducing both the size of the impermeable region and the longitudinal diffusion time. This also explains why after 24h increasing *L*_*p*_ increases the mean oxygen concentration regardless of the initial cell density.

Fig 9B also shows that after 24h the mean cell density increases with *L*_*p*_, independent of the initial cell density. This is consistent with the mean oxygen concentration increasing with *L*_*p*_, and consistent with Fig 7 for a uniformly porous sheath. Still, we note that for the first few hours the variations are slightly more complex due to the high initial oxygen concentration, which tends to favour low initial cell densities when the sheath is impermeable.

Fig 9B also shows that increasing *L*_*p*_ decreases the mean VEGF concentration regardless of time or initial cell density. This is the result of 1) the low VEGF concentration in the porous region, 2) the increase in oxygen concentration in the impermeable region, which hinders VEGF secretion and 3) the decrease of the VEGF longitudinal time of diffusion in the impermeable region.

For *v*_SD_, on the other hand, Fig 9B shows that variations are more complex and non-monotonic. Generally, we see that increasing *L*_*p*_ first increases the standard deviation to a maximum value and then decreases it. When the sheath is completely impermeable (*L*_*p*_ = 0), the mean VEGF concentration is much closer to the maximum of concentration (Fig 3B), which means that the VEGF concentration it is generally distributed close to its mean. Increasing *L*_*p*_ then decreases the mean VEGF concentration, but not so much the maximum VEGF concentration at the centre of the impermeable region, mainly due to slow axial diffusion and limited access to oxygen. This causes the VEGF concentration distribution to spread more, increasing *v*_SD_. Increasing *L*_*p*_ further results in the mean VEGF concentration being closer to the concentration in porous regions and a decrease in the maximum VEGF concentration due to better access to oxygen and shorter longitudinal diffusion in the impermeable region, effectively decreasing *v*_SD_.

### Non-uniform cell seeding strategies

In this section, we investigate the effect of different non-uniform initial cell distributions upon cell survival and VEGF secretion. The premise is that supporting denser populations of cells in specific regions could induce a differential in VEGF secretion across the construct, thereby enhancing VEGF gradients which act as a stimulus for vascular regrowth.

**Fig 10:**
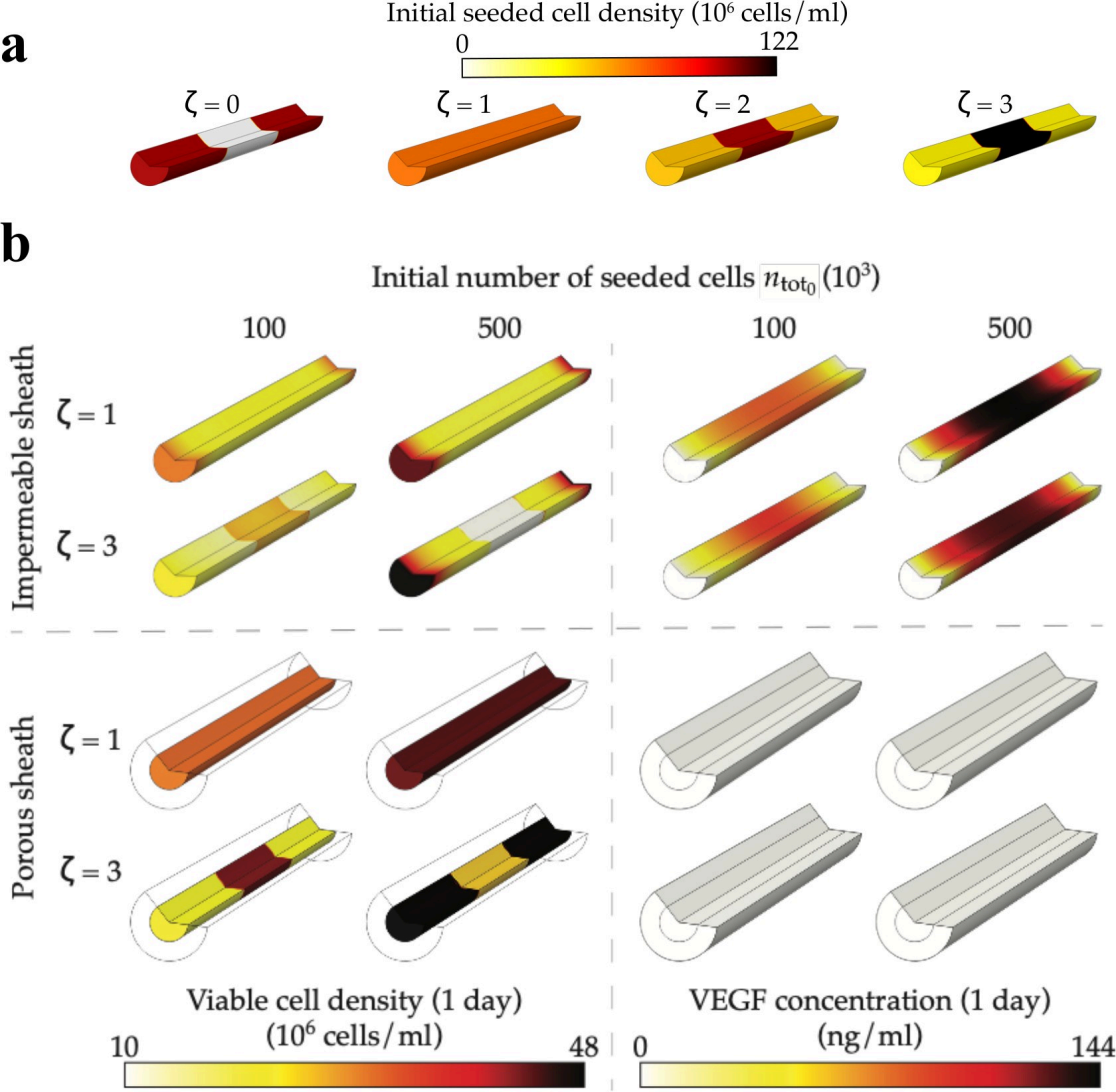
Overconcentrating cells in the centre of the construct leads to lower cell density and VEGF concentration after 24h. **A)** Heatmaps of initial cell density for different repartition factor *ζ* for a constant initial total number of cells *n*_0,total_ = 2×10^5^ cells. The case ζ = 1 corresponds to a uniform distribution. **B)** Heatmaps of cell density (left) and VEGF concentration (right) for impermeable (top) and porous (bottom) sheaths after 1 day. In each panel, simulations are run for two different values of *ζ* and *n*_0,total_. When porous, the sheath structure is approximated by an array of overlapping spheres (see Table 1) with a thickness *T* = 0.25mm and a porosity *ϵ* = 0.8.

To create non-uniformities in the cell distribution we consider the following expression for the initial cell density

$$n_{0}(z)=\left\{ \begin{array}{c} \frac{3n_{0,\mathrm{tot}}}{(2+\zeta )\pi R_{1}^{2}L}\mathrm{if}\;z<\frac{L}{3}\\ \frac{3\zeta n_{0,\mathrm{tot}}}{(2+\zeta )\pi R_{1}^{2}L}\mathrm{if}\;\frac{L}{3}\leq z<\frac{2L}{3}\\ \frac{3n_{0,\mathrm{tot}}}{(2+\zeta )\pi R_{1}^{2}L}\mathrm{if}\;z\geq \frac{2L}{3} \end{array},\right.$$ (46)

where *n*_0,tot_ denotes the initial total number of cells seeded and *ζ* the repartition factor controlling the magnitude of the non-uniformity across 3 equally spaced regions. A small repartition factor (*ζ*<1) concentrates the cells in the regions adjacent to the stumps while a larger one (*ζ*>1) concentrates the cells in the central region of the construct (Fig 10A). Although we focus on dividing the NRC into 3 zones, the approach can be extended. We note that each value of *n*_0,tot_ is related to a uniform cell seeding density *n*_0_ by assuming *ζ* = 1. For instance, *n*_0_≈170, 102 or 34×10^6^cells/ml yield *n*_0,tot_ = 5.00, 3.00 or 1.00×10^5^ cells respectively. Considering the above square function for the initial cell density has two advantages. First, its simplicity will facilitate ease of manufacture. Second, integrating it over the whole construct consistently yields the initial total number of cells, regardless of the repartition factor.

Fig 10B shows heatmaps of cell densities and VEGF concentrations after 24h, for different initial total number of cells, for a uniformly porous and an impermeable sheath, and for uniform (*ζ* = 1) and centre-populated (*ζ* = 3) cases. On the top left panel, the sheath is impermeable, and we see that when *ζ* = 3 and *n*_tot,0_ = 1.00×10^5^cells, the cell density in the central region is higher than in the stump regions. Comparing it with the uniform case, we also see that the cell density in the central region is higher and that the cell densities in the stump regions are lower.

To help interpret such results we compute, for the central and stump regions, the associated initial cell densities. We then compare them to $\mathrm{argma}x_{n_{0}}\bar{n}$, obtained in Fig 3A for a uniform seeding, with the idea that each region can approximately be treated independently so that $\mathrm{argma}x_{n_{0}}\bar{n}$ can serve as a proxy to represent the balance between effects of initial cell density, cell proliferation and cell death. The cell density in the central region for the case *ζ* = 3 and *n*_tot,0_ = 1.00×10^5^cells is *n*_0_≈6.10×10^7^cells/ml, which is closer to $\mathrm{argma}x_{n_{0}}\bar{n}\approx 8.80\times 10^{7}$ cells/ml than the initial cell density in the regions adjacent to the stumps (*n*_0_≈2.00×10^7^cells/ml) or in the uniform case (*n*_0_≈3.40×10^7^cells/ml), explaining the higher cell density in the central region.

By comparison, we see that when *ζ* = 3 and *n*_tot,0_ = 5.00×10^5^cells, the cell density in the central region is much lower. This time *n*_0_≈3.05×10^8^cells/ml in this region, which is relatively far from $\mathrm{argma}x_{n_{0}}\bar{n}$, and hence is associated with low cell density. On the contrary *n*_0_≈1.01×10^8^cells/ml in the stump regions, which is significantly closer to $\mathrm{argma}x_{n_{0}}\bar{n}$ explaining the higher cell density, even compared to *ζ* = 1 (*n*_0_≈1.70×10^8^cells/ml).

The top right panel shows that the corresponding VEGF concentration distributions remain smooth despite discontinuity in the cell density thanks to longitudinal diffusion. Still, we see that when *n*_tot,0_ = 1.00×10^5^cells the VEGF concentration is higher when the cells are concentrated in the central region of the construct. On the contrary, when *n*_tot,0_ = 5.00×10^5^cells the VEGF concentration is higher when the cells are seeded uniformly, although we note that the VEGF concentration still increases between *n*_tot,0_ = 1.00×10^5^cells and *n*_tot,0_ = 5.00×10^5^cells for both cases. Similar to the cell density, such results can be interpreted by comparing to the value of $\mathrm{argma}x_{n_{0}}\bar{v}$ determined in Fig 3B. For instance, *n*_tot,0_ = 5.00×10^5^cells with *ζ* = 1 yields *n*_0_≈1.70×10^8^cells/ml which is closer to $\mathrm{argma}x_{n_{0}}\bar{v}\approx 2.36\times 10^{8}$ cells/ml than any other configuration, explaining the higher VEGF concentration.

The bottom left panel shows that the cell density is globally higher and follows the same pattern when the sheath is porous, regardless of the initial cell density or seeding strategy. This is mainly due to the cells having improved access to oxygen via radial diffusion.

Finally, the bottom right panel shows that when the sheath is porous, VEGF concentrations are very low, regardless of the cell-seeding strategy. This is primarily due to the VEGF radial diffusion time being smaller than the VEGF secretion time (*t*_*v*,R_≈0.31h versus *t*_*v*,P_≥1.65h for the 4 cases considered here) and the cells having improved access to oxygen from the tissue.

**Fig 11:**
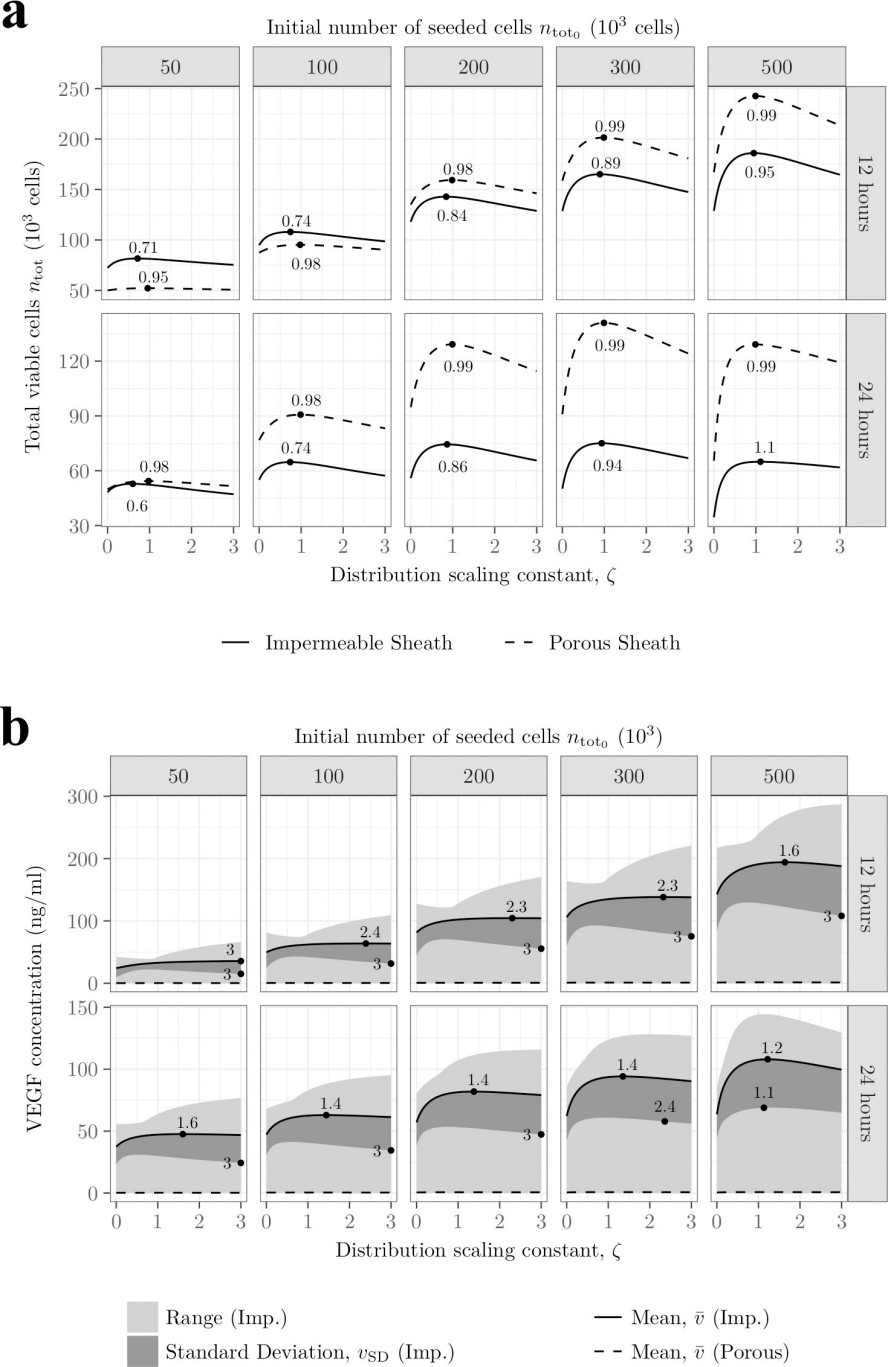
Seeding cells in the stump regions increases cell density and seeding cells in the central region increases VEGF concentration after 24h for an impermeable sheath. **For a porous sheath, uniform seeding leads to maximal cell density**. **A)** Total cell number and **B)** mean, standard deviation (dark grey areas) and ranges (light grey areas) of VEGF concentration, as functions of the repartition factor *ζ* and the initial total number of cells *n*_0,total_, for an impermeable (continuous line) and a porous (dashed line) sheath, after 12 and 24 hours. Black dots indicate the repartition factor *ζ* yielding the highest total number of cells, *i*.*e*. ${argmax}_{\zeta }\;n_{tot},$ the highest VEGF mean concentration, *i*.*e*. ${argmax}_{\zeta }\;\bar{v},$ and the highest standard deviation of VEGF concentration, *i*.*e*. ${argmax}_{\zeta }\;{\bar{v}}_{SD}$. When porous, the sheath structure is approximated by an array of overlapping spheres (see Table 1) with a thickness *T* = 0.25mm and a porosity *ϵ* = 0.8.

Fig 11A extends these results and shows *n*_tot_ after 12h and 24h, as a function of *ζ*, for different $n_{tot_{0}}$ and for a porous and an impermeable sheath.

When the sheath is porous, we see that the total number of cells is non monotonic and maximal for repartition factors close to *ζ* = 1, so that concentrating cells either in the proximal and distal stumps regions or in the central region of the NRC is not beneficial. In this situation the oxygen concentration in the construct is approximately uniform due to quick radial diffusion so that concentrating cells in a specific region only increases the associated initial cell density, which in turn increases the cell death rate. Therefore, uniform seeding remains the best strategy as it allows maximum spreading.

When the sheath is impermeable, on the other hand, we see that concentrating cells in the stump regions is beneficial for small $n_{tot_{0}}$. As the initial cell number increases, however, we see that increasing *ζ*, i.e., the number of cells in the central region, increases *n*_tot_. This is mainly the result of the high initial oxygen concentration that can sustain cell proliferation even when cells are concentrated in a given region. However, seeding too many cells in a specific region, again results in a smaller total number of cells after 24h.

Fig 11A also shows that after 12h, the total number of viable cells is larger when the sheath is impermeable, *i*.*e*. for $n_{tot_{0}}=5.00\times 10^{4}$ cells and for $n_{tot_{0}}=1.00\times 10^{5}$ cells, which are associated with relatively low initial cell densities (*n*_0_≤3.10×10^7^cells/ml for $n_{tot_{0}}=5.00\times 10^{4}$ cells and *n*_0_≤6.20×10^7^cells/ml for $n_{tot_{0}}=1.00\times 10^{5}$ cells when *ζ* = 3). This is again an effect of the high initial oxygen concentration which enhances cell proliferation when the sheath is impermeable. In all other instances, in particular after 24h, the porous sheath exhibits larger total cell numbers because the cells have an improved access to oxygen.

Fig 11A shows the existence of an initial total cell number beyond which the total number of cells decrease after 24h. The precise position of such a maximum slightly depends on the repartition factor and whether the sheath is porous or impermeable, but we see that it is located around $n_{{tot}_{0}}=3.00\times 10^{5}$ cells (equivalent to *n*_0_ = 1.02×10^8^cells/ml). This aligns with values of $\mathrm{argma}x_{n_{0}}\bar{n}$ reported in Fig 3A (8.80×10^7^cells/ml) for an impermeable sheath and in Fig 7 (1.10×10^8^cells/ml) for a porous sheath.

With regard to VEGF, Fig 11B shows the mean, the standard deviation, and the range of VEGF concentration, as functions of the repartition factor *ζ*, for different initial total number of cells and for an impermeable and a porous sheath. Contrary to *n*_tot_, we see that seeding the cells in the central region increases both the mean VEGF concentration and the standard deviation when the sheath is impermeable. The central region is associated with the longest longitudinal diffusion time so that seeding the cells preferentially in this region allows the VEGF concentration to rise to higher values. Still, we note that when the initial total cell number increases it becomes preferable to seed the cells more uniformly. This is the result of the balance between oxygen consumption, cell death and VEGF secretion. When the initial total number of cells is relatively small, the initial concentration of oxygen sustains the cell population longer even if the cells are concentrated in the central region. As the initial total number of cells increases, the oxygen is consumed faster and the cell death rate increases, especially in the central region, which leads to lower cell density, ultimately reducing VEGF secretion. Spreading the cells then increases the consumption time of oxygen while decreasing the cell death rate, sustaining a higher VEGF production.

By comparison, increasing $n_{{tot}_{0}}$ generally results in an increase of the VEGF production after 24h. This is in agreement with Fig 3B which shows that $\mathrm{argma}x_{n_{0}}\bar{v}$ and $\mathrm{argma}x_{n_{0}}v_{SD}^{}$ are significantly larger than $\mathrm{argma}x_{n_{0}}\bar{n}$ and correspond to $n_{{tot}_{0}}$ greater than the values explored in Fig 11B.

After 12h, we see that the maximum of the VEGF concentration range exhibits two maxima (*ζ* = 0 and *ζ* = 3) and one minimum (*ζ*≈1). These two peaks are the results of the cells being concentrated in specific regions. The minimum therefore corresponds to the transition between these two modes of VEGF secretion. We note that after 24h, for $n_{{tot}_{0}}\geq 3.00\times 10^{5}$ cells, this behaviour is reverted, with two minima around *ζ* = 0 and *ζ* = 3 and a maximum value around *ζ* = 1. In this scenario, the oxygen initially present in the seeded region is quickly consumed which results in a quick decay of the associated cell population. Finally, we note that the VEGF standard deviation also follows such behaviour. This is expected as we have shown that *v*_SD_ is controlled by the maximum VEGF concentration (Fig 3B).

Consistent with previous analysis, we see that when the sheath is porous, the mean, standard deviation and range of VEGF concentration are all much lower regardless of the repartition coefficient. This is due to the quick radial diffusion of VEGF and the cells having better access to oxygen, which prevents the VEGF concentration from increasing even when the cells are concentrated in the central region.

Altogether Fig 11 shows that non-uniform seeding does not change dramatically the behaviours described in Figs 3 and 7 for uniform cell seeding.

## Discussion

We devised and simulated a cell-solute model with the goal to comparatively evaluate the short-term performance of a range of NRC designs and to link these outcomes to the underlying transport mechanisms. This approach, using mathematical models informed by experimental data, may identify design strategies for NRCs as well as improve our understanding of the cellular and chemical factors which determine outcomes. Given the challenges and cost of evaluating the full design parameter space using a purely experimental approach, computational modelling may be valuable in refining design choices and informing future experimental work.

Our primary focus was to determine scenarios which promote high cell viability, as well as VEGF concentration and gradients, given that these are key determinants of vascular growth (required to sustain an implanted construct in the longer term). We focused on critical design features which may be controlled when fabricating engineered NRCs: seeded cell density and spatial distribution, and the permeability of the enclosing NRC sheath to molecular factors. The cell-solute model was parameterised against available literature data, including dedicated *in vitro* experiments reported in [12] on a therapeutically-relevant cell type, dADSCs.

Model predictions showed that seeding more cells in a NRC does not necessarily lead to higher cell densities after 24h due to the balance between oxygen transport, cell proliferation and cell death. This result runs contrary to the reasoning generally used in experimental studies, which either neglects to consider the effect of seeding cell densities and distributions entirely or assumes that seeding more cells will necessarily result in a greater number of viable cells over time. In the case of an impermeable sheath, we found that the initial cell density yielding the highest cell density after 24h is $\mathrm{argma}x_{n_{0}}\bar{n}\approx 8.80\times 10^{7}$ cells/ml, which corresponded to less than half the maximal initial cell density tested (Fig 3A). Interestingly, this fell close to the value of 8.00×10^7^cells/ml found by Mosahebi et al. [35] to produce on average the greatest length of axonal regeneration after 3 weeks *in vivo* in a rat sciatic nerve injury model. Increasing the cell density beyond this showed a decrease in regeneration in their experimental study. Guénard et al. [36] produced similar experimental results, also in rat sciatic nerve models, concluding that 1.20×10^8^cells/ml produced the greatest number of myelinated axons after 3 weeks, although in this case the luminal diameter was altered along with the cell seeding density.

We also showed that when the sheath was impermeable $\mathrm{argma}x_{n_{0}}\bar{n}$ was noticeably lower than $\mathrm{argma}x_{n_{0}}\bar{v}$, so that it was not possible to maximise both the VEGF production and the cell density at the same time (Fig 3). We note however that $\mathrm{argma}x_{n_{0}}\bar{v}$ and $\mathrm{argma}x_{n_{0}}v_{SD}$ were still relatively close to each other so that a seeding strategy maximising the mean VEGF concentration also enabled steep VEGF gradients after 24h (Fig 3B).

We further pointed out that for $\mathrm{argma}x_{n_{0}}\bar{n}\approx 8.80\times 10^{7}$ cells/ml the associated cell density was $\mathrm{ma}x_{n_{0}}\bar{n}=2.63\times 10^{7}$ cells/ml, which yielded a relatively small survival rate $\frac{max_{n_{0}}\bar{n}}{argmax_{n_{0}}\bar{n}}\approx 30\%$ after 24h (Fig 3A). Such a cell density is relatively low and may not provide the required support for nerve regeneration over the longer term (*e*.*g*. angiogenesis, neurite growth), or sustain the long-term secretion of VEGF.

We showed that seeding the cell preferentially in the stump regions could help increase the viable cell number after 24h (Fig 11A) and that seeding cells in the centre of the construct on the contrary increased the VEGF concentration and gradients (Fig 11B). We note, however, that in each scenario the optimal repartition factor *ζ* remained close to unity, so that non-uniform seeding strategies did not considerably improve VEGF production or cell density after 24h.

By comparison, increasing the ambient construct oxygen concentration at the outset (via the culture conditions) appeared to be more beneficial, as it resulted in an increase of $\mathrm{ma}x_{n_{0}}\bar{n},\;\mathrm{ma}x_{n_{0}}\bar{v}$ and $\mathrm{ma}x_{n_{0}}v_{SD}$, and the decrease of $\mathrm{argma}x_{n_{0}}\bar{n},\;\mathrm{argma}x_{n_{0}}\bar{v},\;\mathrm{argma}x_{n_{0}}v_{SD}$ (Fig 4A-C). Essentially a high culture oxygen concentration allowed fewer cells to be seeded, which then proliferated well and the viable cell density after 24h was systematically improved along with the secretion of VEGF.

Increasing the initial concentration of VEGF yielded no significative changes to the VEGF field after 24h, mainly due to the range explored being small compared to VEGF concentration originating from cell production. This suggests alternative approaches would be needed to boost VEGF levels in NRCs, for example controlled-release VEGF via biomaterials or nanoparticles. In these cases, the concentration of delivered VEGF should be higher than the range simulated here and could be spatially distributed to augment both VEGF concentration and gradients.

When the sheath was porous, however, the initial concentration of oxygen had little influence on the results due to quick radial diffusion, so that the problem was controlled by the ambient oxygen levels in the surrounding tissue (Fig 7). On one hand, this increased viable cell density after 24h yielding better cell survival ($\frac{max_{n_{0}}\bar{n}}{argmax_{n_{0}}\bar{n}}\approx 45\%$), because cells had improved access to oxygen. On the other hand, this dramatically decreased VEGF concentration and gradients, even when the sheath porosity was relatively small mainly due to quick radial diffusion and the zero VEGF concentration in the tissue, but also because the surrounding tissue concentration of oxygen was above the threshold for VEGF release. Although an option may be to increase the sheath thickness while keeping the sheath porosity relatively low (Fig 8B), this also impaired oxygen availability and resulted in a decrease of viable cell density after 24h (Fig 8A).

A partially porous sheath with an intermediate value for the length of the porous zones (*e*.*g*. 2mm<*L*_*p*_<4mm) appears to address the compromise between sustaining cell viability and stimulating VEGF concentration and gradients (Fig 9B). However, this raises another consideration. Endothelial cells forming the tip of vessel sprouts invading the NRC during angiogenesis will sense VEGF thanks to filopodia extensions that can extend up to 100 microns [74]. A partially porous sheath with a porous zone length of 2-4mm would create a region depleted of VEGF much larger than the range of filopodia, which might hinder endothelial cell invasion and angiogenesis. The balance of these factors should be explored in future experimental work.

Overall, predictions of the computational model indicate that a porous sheath has both advantages and drawbacks. This ambiguity is also present in experimental studies; some report increased neuronal regeneration [75–78] and offer the hypothesis that this could be a result of increased oxygen diffusion and infiltration of supportive cells, whereas others report poorer results when using porous materials [62,79] and suggest this is caused by loss of growth factors and infiltration of host immune cells. We point out however that the model developed in this work is limited to archetypal porous structures and offers only a simplified description of the initial steps of the nerve repair process. Further analysis, combining model predictions with experimental investigation, is required to explore this further.

A way to further assess the benefits of a porous sheath could be to extend simulations beyond the first 24h. The model should then include a description of angiogenic and neurogenic processes taking place on the longer timescale. Similarly, changes in tissue concentration in oxygen and growth factors are likely to happen beyond 24h and should also be included in the model given the importance of the tissue oxygen concentration on determining cell outcomes in the NRC (Fig 5A and 5B). Additionally, an empirical characterisation of the porous structure of the NRC sheath used for EngNT NRC would improve the model physical accuracy. Such an extended model will comprise an array of new parameters, some of which being very hard to estimate as they are not directly measured *in vivo* (*e*.*g*. evolving oxygen concentration in the tissue, endothelial sprouting growth rate) and will require dedicated experiments to narrow down their range.

Finally, an interesting compromise may be a porous sheath with selective properties that take advantage of the difference in molecular weight between VEGF (46kDa for a VEGF dimer [80]) and oxygen (3.2×10^−2^kDa), with pores smaller than VEGF molecules, but larger than oxygen. Such a porous sheath could then prevent loss of VEGF by radial diffusion whilst enabling diffusive transport of oxygen.

In conclusion, the simulation results presented here can be used to discard NRC designs that lead to sub-optimal cell survival and VEGF secretion within the first 24 hours post-implantation. In particular, results indicate that seeding cells beyond a given threshold is detrimental regardless of the situation, that non-uniform seeding is generally not significantly beneficial and that a porous sheath prevents the build-up of a significant VEGF concentration in the NRC. These predictions can now be used to propose new candidate NRC designs for *in vivo* testing. Finally, this study constitutes a first step in laying down the theoretical framework to accelerate the development of treatment strategies for peripheral nerve injuries.
